# Supplementary material for: Cardiac rehabilitation for children and young people (CardioActive): protocol for a single-blind randomised feasibility and acceptability study of a centre-based cardiac rehabilitation programme versus usual care in 11–16 years with heart conditions
Source: BMJ Open. 2024 Feb 24;14(2):e077958. doi: 10.1136/bmjopen-2023-077958 (PMC10895226; doi:10.1136/bmjopen-2023-077958)
Supplement: Author's manuscript [file bmjopen-2023-077958.draft_revisions.pdf]

BMJ Open is committed to open peer review. As part of this commitment we make the peer review history of every article we publish publicly available.

When an article is published we post the peer reviewers' comments and the authors' responses online. We also post the versions of the paper that were used during peer review. These are the versions that the peer review comments apply to.

The versions of the paper that follow are the versions that were submitted during the peer review process. They are not the versions of record or the final published versions. They should not be cited or distributed as the published version of this manuscript.

BMJ Open is an open access journal and the full, final, typeset and author-corrected version of record of the manuscript is available on our site with no access controls, subscription charges or pay-per-view fees (http://bmjopen.bmj.com).

If you have any questions on BMJ Open's open peer review process please email info.bmjopen@bmj.com

BMJ Open

Cardiac Rehabilitation for Children and Young People (CardioActive): Protocol for a Single-Blind Randomized Feasibility and Acceptability Study of a cardiac rehabilitation programme versus usual care in 11-16-year-olds with heart conditions.

Journal:	*BMJ Open*
Manuscript ID	bmjopen-2023-077958
Article Type:	Protocol
Date Submitted by the Author:	31-Jul-2023

Complete List of Authors:	Capobianco, Lora; Greater Manchester Mental Health NHS Foundation Trust,<br>Hann , Mark ; University of Manchester, Centre for Biostatistics and Manchester Academic Health Science Centre<br>McManus, Emma ; The University of Manchester, Health Organisation, Policy and Economics<br>Peters, Sarah; University of Manchester, Health Sciences<br>Doherty, Patrick Joseph; University of York, Health Science<br>Ciotti, Giovanna; Manchester University NHS Foundation Trust, Department of Pedeatric Cardiology<br>Murray, Joanne; Shrewsbury and Telford Hospital NHS Trust<br>Wells, Adrian; The University of Manchester, Division of Psychology and Mental Health; Greater Manchester Mental Health NHS Foundation Trust, Research and Innovation

Keywords:	MENTAL HEALTH, Paediatric cardiology < CARDIOLOGY, REHABILITATION MEDICINE

SCHOLARONE™
Manuscripts

Cardiac Rehabilitation for Children and Young People (CardioActive): Protocol for a Single-Blind Randomized Feasibility and Acceptability Study of a cardiac rehabilitation programme versus usual care in 11-16-year-olds with heart conditions.

Lora Capobianco[1,2], Mark Hann[3], Emma McManus[4], Sarah Peters[2,5,], Patrick Doherty[6], Giovanna Ciotti[7], Joanne Murray[8], Adrian Wells[1,2]

[1] Division of Psychology and Mental Health, School of Psychological Sciences, Faculty of Biology, Medicine and Health, The University of Manchester, Manchester, UK.

[2]Research and Innovation, Greater Manchester Mental Health NHS Foundation Trust, Manchester, UK

[3]Division of Population Health, Health Services Research, and Primary Care, Biostatistics Group, Faculty of Biology, Medicine, and Health, The University of Manchester, Manchester, UK.

[4]Health Organisation, Policy and Economics (HOPE), Division of Population Health, Health Services Research and Primary Care, School of Health Services, Faculty of Biology, Medicine and Health, The University of Manchester, Manchester, UK.

[5]Manchester Centre for Health Psychology, Faculty of Biology, Medicine and Health, Division of Psychology and Mental Health, University of Manchester

[6] Department of Health Sciences, University of York, York, UK

[7] Department of Pedeatric Cardiology, Manchester University NHS Foundation Trust, Manchester, UK

[8] Psychology Service, Shrewsbury and Telford Hospital NHS Trust, Shrewsbury, UK

Corresponding Author: Dr. Lora Capobianco, lora.capobianco@gmmh.nhs.uk

Abstract

Background: Congenital heart conditions are amongst the most common non-communicable diseases in children and young people (CYP), affecting 13.9 million CYP globally. While survival rates are increasing, support for young people adjusting to life with a heart condition is lacking. Furthermore, 1 in 3 CYP with heart conditions also experience anxiety, depression, or adjustment disorder, for which little support is offered. While adults are offered cardiac rehabilitation (CR) to support their mental and physical health, this is not offered for CYP.

One way to overcome this is to evaluate a CR programme comprising exercise with mental health support (CardioActive) for CYP with heart conditions. The exercise and mental health components are informed by the metacognitive model, which has shown to be effective in treating anxiety and depression in CYP and associated with improving psychological outcomes in adult CR.

Method: The study is a single blind parallel randomized feasibility trial comparing a CR programme (CardioActive;CA) against usual care with 100 CYP (50 per arm) aged 11-16 diagnosed with a heart condition. CA will include six group exercise, lifestyle and mental health modules. Usual care consists of routine outpatient management. Participants will be assessed at three time points: baseline, 3-month (post-treatment) and 6-month follow up. Co-primary outcomes (strength and difficulties questionnaire and paediatric quality of life), and a range of secondary outcomes will be administered at each time point. A nested qualitative study will investigate CYP, parents, and healthcare staff views of CR and its components, and staff's experience of delivering CA. Preliminary health economic data will be collected to inform future cost-effectiveness analyses.

Analysis: Descriptive data on study processes and clinical outcomes will be reported. Data analysis will follow intention to treat. Qualitative data will be analysed using thematic analysis and the theoretical framework of acceptability.

For peer review only

Ethics and Dissemination: Ethical approval was granted on 14/02/23 by the Greater Manchester East Research Ethics Committee (REF Ref: 22/NW/0367). The results will be disseminated through peer reviewed journals, conference presentations, and local dissemination.

Trial registration: ISRCTN50031147; NCT05968521

Keywords: cardiac, youth, mental health, metacognition, metacognitive therapy, rehabilitation

Strengths and Limitations of This Study

- The study will evaluate a novel cardiac rehabilitation intervention (CardioActive) for children and young people with heart conditions which includes both mental and physical health support based on evidenced based psychological theory
- Results of the study have the potential to inform a future large scale clinical trial to evaluate the effectiveness and cost effectiveness of the intervention.
- The intervention may aid in bridging the gap between mental and physical health services.

Introduction

Congenital heart disease (CHD) affects 13.8 million children and young people (CYP) under the age of 20 globally [1]. Survivors of CHD face challenges and comorbidities which impact their ability to function [2], future employment, and progression into independent adulthood [3, 4]. CYP with heart conditions have reduced quality of life and poorer psychological functioning [5-7, as 41% of CYP with CHD experience significant difficulties adjusting to their heart condition, and 30% experience clinically significant anxiety or depression [7-13]. In addition, overprotection from parents, educators, and healthcare staff contribute to reduced physical activity, and exercise intolerance [14-17]. This is particularly important as sedentary lifestyles are a risk factor for atherosclerosis, cardiovascular disease, obesity and diabetes [18].

A possible means of improving physical and mental health outcomes would be to facilitate access to cardiac rehabilitation (CR) that improves healthy behaviours such as exercise and psychological self-

For peer review only

management skills. CR programmes provide a unique opportunity to improve physical health, provide education around lifestyle and risk factors, and provide psychological support [19,20]. In the UK, following a cardiac event, adults are encouraged to attend CR programmes, which have been recommended by the National Health Service (21), the Department of Health, National Institute for Health and Care Excellence (22) guidelines (CG172, CG94 and NG106) and the British Association for Cardiovascular Prevention and Rehabilitation (23). However, such programmes are not routinely available for CYP living with heart conditions.

In adults CR programmes have been found to reduce patient mortality, morbidity, hospital re-admissions, improve quality of life, and be a cost-effective intervention [24, 25]. This is despite previous systematic reviews and meta-analyses indicating that paediatric CR has been shown to improve physical, developmental, cognitive, and psychosocial outcomes [24, 26-28]. Akamagwuna and Badaly [24] conducted a systematic review of 20 paediatric cardiac rehabilitation programmes. They found that both home-based and outpatient programmes were associated with improvements in cardiorespiratory outcomes, quality of life and psychosocial functioning.

Previous youth CR programmes have predominantly focused on improving physical health outcomes, with less attention given to mental health components, despite 41% of patients struggling with adjusting to living with a heart condition. Tesson et al [29] conducted a systematic review of psychological interventions for childhood-onset heart disease and found only two studies focused on interventions for CYP [30,31]. However, the results were disappointing, as they did not lower anxiety, depression [31] or improve quality of life [30]. CR with CYP might clearly benefit from the use of routine exercise programmes that can be coupled with psychological input aimed at improving adjustment and mental health outcomes.

Drawing on the literature on physical and psychological effects of CR techniques in CYP [29-32] and informed by theory and research on metacognitive therapy in adult CR and mental health [33-35]), we have devised CardioActive (CA), a 6-session CR package for use with CYP. The physical health

For peer review only

component has been informed by recommendations for physical activity in CYP with CHD (36-39), and the latest systematic reviews and meta-analyses which suggest that programmes should include a combination of endurance and strength-based exercises (29-32). The mental health/adjustment component of CA is informed by the metacognitive model of anxiety, depression and stress and focuses on helping CYP to reduce their worry and unhelpful self-monitoring processes. The approach has been usefully applied in adult CR to improve psychological outcomes.

While MCT is promising, further research is needed to understand the views and responses to MCT delivered as part of CR for CYP. As such the aim of the proposed study is to develop a cardiac rehabilitation programme (CardioActive) which incorporates mental health support for CYP and to evaluate the acceptability and feasibility of the programme in comparison to usual care.

Methods

Design

The current study is a parallel single-blind randomised feasibility study in children and young people aged 11-16 with a heart condition with 12 and 24 weeks follow up, comparing) Routine clinical management (treatment as usual (TAU)) against the CardioActive (CA) programme. Qualitative investigation will be embedded within the trial. Preliminary economic data will be obtained to inform the optimum way of evaluating cost-effectiveness in any subsequent definitive trial. See Figure 1 for an overview of the trial design according to CONSORT guidelines [40] [41]. The recommendations for the Interventional Trials 2013 (SPIRIT) Checklist [42] [43] is included in Additional file 1. Figure 2 outlines the schedule of recruitment, interventions, and assessment.

INSERT FIGURE 1 HERE

INSERT FIGURE 2 HERE

For peer review only

Trial population

The trial population are children and adolescents attending cardiology outpatient services.

Eligibility Criteria

In order to be eligible to take part in the study participants will meet the following inclusion and exclusion criteria:

Inclusion Criteria

1. Aged between 11-16 years.
2. Ability to consent to participate.
3. Native fluency in English language.
4. Diagnosed with at least one of the following: congenital heart disease (all subtypes), cardiomyopathy, cardiac arrhythmia, heart failure, post-cerebrovascular event, post-heart valve repair/replacement.

Participants will be excluded if they meet one or more of the following:

1. Presence of significant risk or safeguarding concerns
2. Head injury/organic impairment
3. Significant communication and/or social difficulties

Only patients with a formal diagnosis or under assessment for one of the exclusion criteria will be excluded from the study.

Recruitment and allocation

Participants will be screened for eligibility during routine clinic appointments by clinicians and/or cardiac team members. Clinicians/cardiac team members will ask eligible patients and their parent/caregiver if they are interested in hearing more about the research study and if

For peer review only

their contact details can be passed onto the research team. The research team will then contact eligible and interested patients and their caregivers to provide further information about the study, answer any questions, arrange for consent to be obtained, and collect baseline data. Once baseline data have been collected, participants will be randomised to a study arm using sealed-envelope software [44]. Randomisation (at the individual level) will be independent and concealed, after stratification by age (11-13; 14-16) and sex (male; female). Within strata, allocation will occur within blocks of size 4 or 6, determined at random and with equal probability by the software. This will minimise future allocation 'predictability'. A member of the research team not blinded to allocation will contact parents/caregivers to inform them of the allocation within 2 days. Blinding of allocation will be maintained for quantitative research assistants, and the trial statistician until all outcome measures for all subjects have been collected. The Trial Executive Committee (TEC) will regularly monitor any unblinding's and corrective action will be implemented if needed.

Trial Conditions

CardioActive Programme

The programme will consist of six sessions lasting up to 90 minutes of a structured exercise programme with integrated educational and lifestyle modules. Exercise sessions have been reviewed by a physiotherapist with experience working with patients with heart conditions and a child cardiologist who have deemed the exercise safe for CYP to engage in. Patients are free to stop the exercise if they begin to feel unwell. Lifestyle modules will focus on modifiable risk factors. The duration of the CR programme is briefer than previous programmes whereby patients receive approximately 2.5 sessions per week for 16 weeks [24, 26-28, 32]. The psychological treatment component (Metacognitive Therapy) is integrated throughout each session and focusses on controlling worry and using adaptive attention strategies. Sessions

For peer review only

include group discussions, experiential learning and homework tasks that participants are asked to complete between sessions.

Treatment as Usual (TAU)

TAU will consist of routine clinical outpatient management, routine screening, and medication management, which includes a review with their cardiologist or cardiac specialist nurse every 6 to 12 months, depending on the nature and severity of the heart condition.

Staff training and supervision

Staff from the cardiology department will receive two full training days in the intervention followed by six weeks of supervised practise in delivering the intervention to a pilot group of volunteers. A further workshop will address any challenges the staff's experienced when delivering the intervention. Staff's delivery of the intervention will be monitored by listening to audio-recordings of sessions and they will receive supervision throughout the trial. Adherence to the intervention will be assessed using an adherence checklist completed by the staff at each session.

Data collection

There will be three assessment points: baseline, 12 weeks (end of treatment), and 24 weeks (follow-up). To ensure completeness of the questionnaires, participants will be asked to arrange a visit, either at an NHS stie or in their own home, at each timepoint. Participants total time involvement in the study will be 24 weeks. Participants will be reimbursed with a £10 shopping voucher for completing each assessment.

Criteria for discontinuation

Participants may withdraw from the study at any time, without any consequences to themselves, their health care, or their ability to take part in future research. Participants who withdraw will continue to receive usual care. Participants can also be withdrawn at the request

For peer review only

of the Chief Investigator or cardiology service, but this would only happen if a participant's life or long-term health or welfare was at risk from continued participation in the study.

Outcomes

The primary outcomes for the study are feasibility and acceptability outcomes. Feasibility will be assessed using referral rates, recruitment and retention rates, attendance at the intervention, rate of return and level of completion of follow up data and willingness to be randomized to treatment. Qualitative focus groups or interviews, as preferred, will be used to understand patients' views of acceptability of the intervention. All outcomes listed below will be obtained at each assessment time point.

Measures

Strength and Difficulties Questionnaire (SDQ; [45]).

The SDQ is a behavioural measure completed by the caregiver for children aged 2 to 17, across five subscales: emotion, hyperactivity, conduct, peer relations and pro-social behaviour. Each subscale contains five items, and each item is scored "Not True", "Somewhat True" or "Completely True". Scores for the subscales range from 0-10, with total difficulties score (0 – 40) generated by summing together the scores from all subscales except pro-social. Higher scores on the pro-social scale reflect strengths whereas higher scores across other subscales reflect difficulties [45]. The SDQ subscales have satisfactory internal consistency (mean Cronbach's alpha = 0.70) and test-retest reliability ranging from 0.75 – 0.91 [46].

Paediatric Quality of Life (PedsQol: [47]).

The PedsQol consists of 23 items which measures health related quality of life including physical, emotional, social, and school functioning, and is commonly used in paediatric cardiology [5, 48]. The items are scored on a Likert scale ranging from 0 (Never) to 4 (Almost

For peer review only

always). Total scores range from 0-100, with higher scores indicating better quality of life. The PedsQol will be completed by CYP.

Six minute walk test (6MWT: [49]).

The 6MWT is used to assess aerobic capacity and endurance. Participants are asked to walk as far as possible for six minutes and the total distance covered during this time is used as the outcome. Performance on the 6MWT has been predictive of morbidity and mortality whereby poorer performance is associated with increased mortality [49].

Incremental Shuttle Walk Test (ISWT: [50])

The ISWT is used to assess exercise capacity. The ISWT is a 12-level test (1 min in each level), whereby participants walk up and down a 10 m course with increasing acceleration of 0.17 m/s up to a maximum speed of 2.37 m/s. The walking speed is dictated by an audio signal. The test ends when the subject has a heart rate greater than 85% of their predicted maximum, is limited by dyspnoea, or when the subject is unable to maintain the required speed and does not complete a shuttle for a second consecutive time. The distance covered from the number of shuttles will be calculated.

Physical Activity Monitoring.

Participants will wear an accelerometer (Actigraph) for five days to monitor physical activity levels at each assessment.

Metacognition Questionnaire-Adolescent (MCQ-A; [51]).

The MCQ-A assesses metacognitive beliefs (beliefs about thinking) across five subscales: uncontrollability and dangerousness of worry, need to control, cognitive self-consciousness, positive beliefs about worry, and cognitive confidence. The 30 items are scored on a Likert scale from 1 (do not agree) to 4 (agree very much) with total scores ranging from 30-120 and 6-30 for each subscale [51]. The internal consistency of the MCQ-A across total scores and most

For peer review only

subscales have been supported with adequate to excellent Cronbach alphas (0.76 – 0.92) [52]. This MCQ-A will be completed by CYP.

Child Health Utility-9D (CHU-9D: [53])

The CHU-9D measures paediatric quality of life using nine dimensions (including worried, sad, pain, tired, annoyed, schoolwork/homework, sleep, daily routine, and ability to join in activities) and is suitable for CYP aged between 7–17-years-old. Each item has 5 levels ranging from no problems to inability to do the item [53]. The CHU-9D has strong internal consistency (Cronbach's alpha = 0.79) and strong test-retest reliability (Interclass correlation coefficients = 0.75) [54]. The CHU-9D will be completed by CYP.

Demographic information questionnaire

The demographic questionnaire will collect variables including child's age, sex, weight, height, BMI, type of heart problem, comorbid mental and physical illnesses, ethnicity, medication, socioeconomic status, parental occupational status. This will be completed by parents/primary caregivers.

Health and Social Care Service-Use Questionnaire (SUQ)

The SUQ will include questions about whether the child has used any primary, secondary, or community-based health and social care and how often they used the service in the last 16 weeks (baseline study visit) or since the last assessment (follow-up study visits). The questionnaire will be developed from existing child relevant service use questionnaires held by the co-applicants and through discussion with the PPI representative, parent advisory group and clinical members of the study team. This will be completed by parents/primary caregivers.

Qualitative Nested Study

We will investigate patients', caregivers', and healthcare professionals' experiences of the CardioActive programme, targets for improvement, perceived acceptability of components of

For peer review only

CR, and examine participants experience of living with/managing heart conditions using semi-structured interviews with CYP (n = 20), caregivers (n = 10), and healthcare professionals (n = 10). Sampling will be purposive to ensure variation in key variables including age, type of health condition, relationship to CYP, and discipline of health care professional (e.g., physiotherapist, cardiologist, clinical psychologist etc). We will endeavour to recruit patients with a range of experiences of CR including those for whom engagement has been low as well as those who have attended regularly. We will explore the acceptability of CR, factors influencing participants' engagement, and barriers and enablers to CR being implemented within services. We will also explore with participants their experiences of key trial processes (e.g., outcome measures, recruitment, and randomisation procedures) to inform the design of a definitive trial. Members from the PPIE group will be involved in the development of the interview schedule and piloting for the qualitative interviews. Participants will be reimbursed for taking part in qualitative interviews or focus groups.

Sample size calculation

Feasibility trials are not powered to provide a definitive effectiveness analysis. Therefore, the sample size is based on having sufficient patients to evaluate the acceptability/feasibility of the intervention (as measured by patient acceptability and adherence ratings, recruitment, and retention rates), and to obtain a provisional estimate of the 'promise' of the intervention (e.g. an 80% C.I.) for powering a future definitive RCT. We will recruit 50 patients per arm (total N = 100), which would allow for an overall attrition rate of up to 20% to be determined to within eight absolute percentage points with 95% confidence. The total sample size is in line with recommendations by Lancaster et al [55]. This will also be sufficient to estimate key 'clinical' parameters, such as the standard deviation of potential outcomes (in the larger trial), with adequate precision, for which samples of 40 are generally sufficient [56].

For peer review only

Our qualitative sample has been informed by prior experience and published guidance [57]. Previous research suggests that a sample size of 12 can achieve data saturation [58]. The exact number will be determined by data sufficiency, achieving adequate variance in recruitment and thematic saturation, however we are confident this estimate will be sufficient to answer the relevant research questions.

Analyses

Quantitative analyses

As this is a feasibility study, we will not be carrying out hypothesis testing to determine if the intervention is effective. Instead, descriptive data on study processes and clinical outcomes will be reported. Following an Intention-To-Treat (ITT) protocol, we will present data relating to participant recruitment, randomisation, retention at follow up and drop out rate in a CONSORT flow chart. The attrition rate will be calculated (with a 95% confidence interval) and reasons for drop-out will be recorded where possible. We will also assess rates of missing data (including which elements) on specific questionnaires and whether any of the measures display floor and/ or ceiling effects.

We will summarise by trial arm, as appropriate (e.g., mean/ standard deviation (SD); median/ inter-quartile range; proportion/95% confidence interval; data range), data for all potential (i.e. for the larger trial) clinical primary outcome measures. We will also report 80% confidence intervals for the trial arm co-efficient from appropriate regression analyses (adjusting for age, gender and the corresponding baseline outcome) by way of investigating the promise of the CardioActive program. This information, the SD of the SDQ and the PedsQoL, the estimated attrition rate and recruitment-based data will be used to help inform the sample size calculation for the large-scale, definitive RCT (in addition to other (published) sources). The statistical analysis plan will be produced by MH (statistician) prior to the examination of outcomes.

Qualitative analyses

For peer review only

All focus groups and interviews will be transcribed verbatim. Thematic Analysis [59] and the theoretical framework of acceptability [60] will be used, allowing for both inductive and deductive coding. Members from the PPIE group will provide feedback on emerging analyses.

Economic analyses

We will evaluate the feasibility of conducting an economic evaluation of CR, by exploring the costs and outcomes associated with CR compared to standard care. We will co-design a health-resource use questionnaire with our PPIE group to capture any changes in healthcare resource use during the study, which will be completed by CYP and their guardians. We will explore resource use from the perspective of the NHS and personal social services, as well as taking a wider perspective to examine if there are any patient out-of-pocket expenses. We will also examine the resources and their associated costs required to develop and deliver the intervention.

We will measure health outcomes using two measures, as described above: the CHU-9D and PedsQol [61]. The CHU-9D has a published value set in which to generate utility scores [53] and therefore quality adjusted life years. There is also a published mapping algorithm to transform the PedsQol to the EQ-5D [61] [62]. We will examine the acceptability of both measures to the CYP and assess data completeness, to inform which questionnaire is most appropriate for use in the definitive study, or if indeed, another measure of paediatric quality of life would be more appropriate.

We will conduct a preliminary economic evaluation using the data collected, to determine whether CR plus TAU would be cost-effective from the perspective of the NHS and Personal Social Services.

Multiple imputation and censored data analysis techniques will be used to separately impute missing observations from participants who complete follow-up, and missing follow-up data for participants lost to follow-up. The primary and sensitivity economic analyses will be controlled

For peer review only

for key baseline covariates or characteristics. Cost effectiveness acceptability curves will assess the likely cost effectiveness of the intervention and uncertainty in the observed data. This cost-effectiveness analysis will be limited due to the sample size and the overall health economic work will focus on informing a definitive trial.

Trial management and oversight arrangement

The trial is managed by a Trial Executive Committee (TEC) which includes those individuals responsible for the day-to-day management of the study including the chief investigator, co-investigators and identified collaborators, principal investigators, and the study statistician. Notwithstanding the legal obligations of the Sponsor and Chief Investigator, the TEC has operational responsibility for the conduct of the study including monitoring overall progress to ensure the protocol is adhered to and to take appropriate action to safeguard the patients and the quality of the study. There will also be patient and public involvement groups (adult and CYP) which will meet at least quarterly and provide advice and feedback on a range of trial-related activities, e.g., reviewing study documents. The end of the study will be reported to the REC within the required timeframe if the study is terminated prematurely. Investigators will inform patients of any premature termination of the study and ensure that the appropriate follow up is arranged for all involved. Following the end of the study a summary report of the study will be provided to the REC within the required timeframe.

For peer review only

Data management

Participants will be allocated a study identity code number for use on all study documents and the electronic database. The study team will make a separate confidential database for the participant's name, date of birth and study identity code to permit identification of participants enrolled in the study e.g., for follow-up. All other information will be anonymised. All study documents (including participant's written consent forms) which are to be held at the participating centres will be held in strictest confidence. Access to patient information shall be restricted to

authorized persons. Data will only be available through restricted, shared areas on the secure GMMH and University of Manchester computer systems (password and username secured). Baseline and follow-up data, which is anonymous data, will be stored in locked filing cabinets at GMMH. These data will be entered into an electronic database for analysis purposes by study team members blind to trial arm allocation. All computers are password protected and adhere to the secure storage policies of the NHS trust and University of Manchester.

Safety reporting

Adverse events (AEs) and serious adverse events (SAEs) will be monitored throughout the study. Any adverse events identified as likely to be caused by the intervention during the period of recruitment or intervention on the study will be recorded at the study site using an AE/SAE record form which will be completed by the health professionals. Study RA and study PIs will be informed, and information inputted onto a secure database. The PIs will also assess if the nature of the event is likely to have been induced because of the study intervention or processes. Any that are deemed be serious and related to the intervention will be reported to the Sponsor, the study executive committee, and ethics committee within 24 hours of the event.

Dissemination and publication policy

The main study results will be published in the name of the study in a peer-reviewed journal, on behalf of all collaborators. The manuscript will be prepared by a writing group, appointed from amongst the TSC. All participating centres and clinicians will be acknowledged in this publication. All presentations and publications relating to the study must be authorised by the study executive committee and sponsor, on whose behalf publications should usually be made. Authorship of any secondary publications resulting from the intervention will reflect the intellectual and time input into these studies and will not be the same as on the primary publication. No investigator may present or attempt to publish data relating to this study without prior permission from the TSC. The

For peer review only

findings will also be presented at national, international, and regional conferences and in public involvement events where the information from this study is relevant.

Patient and Public Involvement

A youth PPI group comprised of young people with lived experience of a heart condition (with and without mental health difficulties) has been created along with an adult PPI group comprised of parents, caregivers, and adult heart condition patients (with and without mental health difficulties). Both PPI groups have aided in the development of the CardioActive programme, in obtaining ethical approval, co-developed patient facing materials, helped to develop the patient recruitment and retention plan, have helped to develop the study name and logo, and will further assist with retention, development of the dissemination plan, and with dissemination of research findings.

Discussion

CHD presents as one of the most common non-communicable childhood diseases. While medical advances have improved survival rates, young people with heart conditions continue to face significant challenges, comorbidities, reduced quality of life and increased psychological distress. NHS England has highlighted that cardiac services must be provided in a way that allows the best outcomes for all patients, not only in reducing mortality, but also in decreasing disability and improving opportunities to lead better lives [63].

A CR programme that integrates mental and physical healthcare has the potential to meet NHS objectives [64] which aims to ensure that CYP, their family and carers have an understanding of the patient's condition, its impact on their health and future, and an understanding of the condition's psychological, social, and cultural factors in addition to service specifications for cardiac disease [65].

The present study would meet service standards and fits within current priorities for closer integration of psychological and physical health services [19,20,66]. Development and evaluation of

For peer review only

a CR programme is an important platform for the progression of young person's cardiac services and will provide valuable qualitative and quantitative data to support the design of a future large-scale definitive trial to test the effectiveness of CardioActive for CYP with CHD.

Declarations

Ethical approval

The study received full ethical approval on February 14, 2023, from North West – Greater Manchester East Research Ethics Committee, Research Ethics Committee (REC) REC ref: 22/NW/0367, IRAS ID 319134. The trial has been registered with clinicatials.gov (NCT05968521) and with ISRCTN (ISRCTN50031147). The study protocol used was Version 6: 21/06/2023. Any modifications to the trial will be submitted for further ethical approval and approved changes will be documented and communicated to the REC, trial registry, executive committee, and all relevant parties. The study will be conducted in accordance with the ethical principles that have their origin in the Declaration of Helsinki (1996), the principles of Good Clinical Practice, and the UK Policy Framework for Health and Social Care Research (2017). GMMH acts as the sponsor for this study. As the sponsor is an NHS organisation, the NHS indemnity scheme will apply. Participating sites will be liable for clinical negligence and other negligent harm to participants taking part in the study and covered by the duty of care owed to them by the sites concerned.

Written informed consent will be obtained from all participants and their parents/caregivers. Participants will be free to withdraw from the study at any time without providing a reason or their care being impacted. All the information collected during this trial will be confidential and held in accordance with NHS Data Protection guidelines and Good Clinical Practice guidelines. Confidentiality will only be breached if participants disclose information which may indicate that there is a risk of harm to themselves or others. Every opportunity to discuss any possible breaches of confidentiality with participants will be taken prior to informing any appropriate agencies, e.g., CAMHS staff, GP or A&E services.

For peer review only

All researchers and study site staff involved with the study must comply with the requirements of the Data Protection Act 1998 regarding the collection, storage, processing, and disclosure of personal information, and will uphold the Act's core principles. Audio-recordings and transcriptions of interviews will be stored on NHS drives, which are password protected and designed for the storage of confidential research material. Interviews which are transcribed will be anonymised at the point of transcription. Any third party involved with transcribing of interviews will sign a confidentiality agreement and be fully instructed in how to anonymise transcripts.

Author Contributions

LC is the chief investigator and conceived the study, designed, and developed the initial trial protocol, developed the trial intervention, and contributed to the first and subsequent drafts of the manuscript. AW is the co-chief investigator and contributed to the study conception, designed, and developed the initial trial protocol, developed the trial intervention, and contributed to the first and subsequent drafts of the manuscript. AW provided translational guidance in applying the metacognitive model in CR. MH contributed to the study design, statistical analysis, and sample size and wrote the quantitative evaluation section of the manuscript. EM developed the health economics components of the protocol and wrote these sections of the manuscript. GC contributed to the development of the trial recruitment strategy. PD contributed to study design. JM contributed to adverse event reporting. SP developed the qualitative components of the protocol and wrote these sections of the manuscript. All authors edited the manuscript and read and approved the final version.

Consent for publication

Not applicable.

Availability of data and materials

The datasets generated and/or analysed during the current study are not publicly available

For peer review only

due to ongoing data collection but are available from the corresponding author on reasonable request on completion of data collection and data analysis.

Competing interests

AW is the creator of Metacognitive Therapy, and co-director of the MCT Institute.

Funding

The study is funded by the National Institute for Health Research (NIHR) under its Research for Patient Benefit Grant (Grant Reference Number: NIHR203634). The views expressed are those of the author(s) and not necessarily those of the NIHR or Department of Health.

For peer review only

References

1. Children & Non-Communicable Disease: Global Burden Report 2019 – NCD Child. (2019). Retrieved 4 May 2022, from https://www.ncdchild.org/2019/01/28/children-non-communicable-disease-global-burden-report-2019/
2. Cooper DS, Jacobs JP, Chai PJ, Jaggers J, Barach P, Beekman RH, et al. Pulmonary complications associated with the treatment of patients with congenital cardiac disease: consensus definitions from the multi-societal database committee for pediatric and congenital heart disease. Cardiol Young. 2008; 18 Suppl 2:215–221.
3. M. Kamphuis, T. Vogels, J. Ottenkamp, E.E. van der Wall, S.P. Verloove-Vanhorick, H.W. Vliegen. Employment in adults with congenital heart disease. Arch Pediatr Adolesc Med. 2002; 156(11):1143-1148
4. Brandhagen DJ, Feldt RH, Williams DE. Long-term psychologic implications of congenital heart disease: a 25-year follow-up. Mayo Clin Proc. 1991; 66(5):474-479
5. Vigl M, Niggemeyer E, Hager A,Schwedler G, Kropf S, Bauer U. The importance of socio-demographic factors for the quality of life of adults with congenital heart disease. Qual Life Res Int J Qual Life Asp Treat Care Rehab. 2011; 20(2):169-177.
6. Moons P, Van Deyk K, Marquet K, De Bleser L, De Geest S, Budts W. Profile of adults with congenital heart disease having a good, moderate, or poor quality of life: a cluster analytic study. Eur J Cardiovasc Nurs. 2009; 8(2):151-157.
7. Latal, S. Helfricht, J.E. Fischer, U. Bauersfeld, M.A. Landolt. Psychological adjustment and quality of life in children and adolescents following open-heart surgery for congenital heart disease: a systematic review. BMC Pediatr. 2009; 9(6).
8. Abda A, Bolduc ME, Tsimicalis A, Rennick J, Vatcher D, Brossard-Racine M. Psychosocial outcomes of children and adolescents with severe congenital heart defect: a systematic review and meta-analysis. J Pediatr Psychol. 2019; 44:463-477.

For peer review only

9. Wang QF, Hay M, Clarke D, Menahem S. The Prevalence and Predictors of Anxiety and Depression in Adolescents with Heart Disease. Journal of Pediatrics. 2012; 161(5):943-946.

10. Awaad MI, Darahim KE. Depression and anxiety in adolescents with congenital heart disease. Middle East Current Psychiatry. 2015; 22,2-8.

11. Gonzalez, V.J., Kimbro, R.T., Cutitta, K.E., Shakosky, J.C., Bilal, M.F, Penny DJ, Lopez KN. Mental Health Disorders in Children With Congenital Heart Disease. Pediatrics February. 2021; 147(2):e20201693. doi: 10.1542/peds.2020-1693

12. Anton CM, Drake MB, Butts RJ, Cao T, Rezaeizadeh A, Trivedi MH, et al. Electronic Mental Health Screening in a Pediatric Heart Failure and Transplant Clinic. J Clin Psychol Med Settings. 2021; 28(4):815-825. doi:10.1007/s10880-021-09770-8

13. Longmuir PE, Sampson M, Ham J, Weekes M, Patel BJ, Gow RM. The mental health of adolescents and pre-adolescents living with inherited arrhythmia syndromes: a systematic review of the literature. Cardiol Young. 2018; 28(5):621-631. doi:10.1017/S104795111700289X

14. Belardinelli R, Georgiou D, Cianci G, A. Purcaro A. Randomized, controlled trial of long-term moderate exercise training in chronic heart failure: effects on functional capacity, quality of life, and clinical outcome. Circulation. 1999; 99(9):1173-1182

15. Casey FA, Stewart M, McCusker CG, Morrison ML, Molloy B, Doherty N, et al. Examination of the physical and psychosocial determinants of health behaviour in 4–5-year-old children with congenital cardiac disease. Cardiol Young. 2010; 20:532–537.

16. Linde LM. Psychiatric aspects of congenital heart disease. Psychiatr Clin North Am. 1982; 5:399–406.

17. Reybrouck T, Mertens L, Physical performance and physical activity in grown-up congenital heart disease. Eur J Cardiovasc Prev Rehabil. 2005; 12(5):498-502

18. Thijssen DH, Maiorana AJ, O'Driscoll G, Cable NT, Hopman MT, Green DJ. Impact of inactivity and exercise on the vasculature in humans. Eur J Apply Physiol. 2010; 108:845-875.

For peer review only

19. Das P, Naylor C, Majeed A. Bringing together physical and mental health within primary care: a new frontier for integrated care. J R Soc Med. 2016; 109:364–6.doi:10.1177/0141076816665270

20. Firth J, Siddiqi N, Koyanagi Ai, Siskind D, Rosenbaum S, Galletly C, et al. The Lancet Psychaitry Comission: A Blueprint for Protecting Physical Health in People with Mental Illness. The Lancet Psychiatry. 2019; 6(8),675-712.

21. National Health Service (NHS). The NHS Long Term Plan. 2019. Available from: https://www.longtermplan.nhs.uk/wp-content/uploads/2019/08/nhs-long-term-plan-version-1.2.pdf

22. National Institute for Health and Care Excellence (NICE). Chronic heart failure in adults: diagnosis and management. NICE guideline 106. 2018. Retrieved from: https://www.nice. org.uk/guidance/ng106

23. British Association for Cardiovascular Prevention and Rehabilitation (BACPR). BACPR Standards and Core Components (3rd Edition). 2017. Retrieved From: http://www.bacpr.com/resources/6A7_BACR_Standards_and_Core_ Components_2017.pdf

24. Akamagwuna, U., Badaly, D. Pediatric Cardiac Rehabilitation: a Review. Curr Phys Med Rehabil Rep. 2019; 7:67–80. https://doi.org/10.1007/s40141-019-00216-9

25. Anderson L, Thompson DR, Oldridge N, Zwisler AD, Rees K, Martin N, et al. Exercise-based cardiac rehabilitation for coronary heart disease. Cochrane Database Syst Rev, 2016; 1:CD001800. doi: 10.1002/14651858.CD001800.pub3

26. Gomes-Neto M, Saquetto MB, Silva CM, Conceicao CS, Carvalho VO. Impact of Exercise Training in Aerobic Capacity and Pulmonary Function in Children and Adolescent After Congenital Heart Disease Surgery: A Systematic Review with Meta-analysis. Pediatr Cardiol. 2016; 37(2):217-24.

27. Duppen N, Takken T, Hopman MTE, Ten Harkel ADJ, Dulfer K, Utens EMWJ, et al. Systematic review of the effects of physical exercise training programmes in children and young adults with congenital heart disease. Int Jnl Cardiology. 2013; 168(3):1779-87.

For peer review only

28. Tikkanen AU, Oyaga AR, Riano OA, Alvaro EM, Rhodes J. Paediatric cardiac rehabilitation in congenital heart disease: a systematic review. Cardiology in the young. 2012; 22:241-250.

29. Tesson S, Butow PN, Sholler GF, Sharpe L, Kovacs AH, Kasparian NA. Psychological interventions for people affected by childhood-onset heart disease: A systematic review. Health Psychology. 2019; 38(2),151–161. Available from: doi:10.1037/hea0000704

30. Lee S, Lee J, Choi JY. The effect of a resilience improvement program for adolescents with complex congenital heart disease. European Journal of Cardiovascular Nursing, 2017; 16:290–298. Available from: http://dx.doi.org/10.1177/1474515116659836

31. Freedenberg VA, Hinds PS, Friedmann E. Mindfulness-based stress reduction and group support decrease stress in adolescents with cardiac diagnoses: A randomized two-group study. Pediatric Cardiology. 2017; 38:1415–1425. Available from: doi:10.1007/s00246-017-1679-5

32. Capobianco L, Adewusi J, Cooper B, Belcher A, Wells A. Effectiveness of physical and mental health interventions for young people with heart conditions: protocol for a systematic review and meta-analysis. BMJ open. 2023 Feb 1, 13(2):e067342.

33. Wells, A. Metacognitive Therapy for Anxiety and Depression. 2009. New York, NY; London: Guilford Press.

34. Wells A, Reeves D, Capobianco, L. Heal C, Davies L, Heagerty AM, Doherty P, Fisher PL. The effectiveness of psychological interventions for depression and anxiety in cardiac rehabilitation: PATHWAY- a single-blind parallel randomised controlled trial of group metacognitive therapy. Circulation. 2021, 144(1), 23-33. Available from: https://doi.org/10.1161/CIRCULATIONAHA.120.052428

35. Normann N, Morina N. The Efficacy of Metacognitive Therapy: A Systematic Review and Meta-Analysis. Front Psychol. 2018, 14(9),2211. Available from: doi:10.3389/fpsyg.2018.02211

36. Tran D, Maiorana A, Ayer J, Lubans DR, Davis GM, Celermajer DS, d'Udekem Y, Cordina R. Recommendations for exercise in adolescents and adults with congenital heart disease. Progress in cardiovascular diseases. 2020 May 1, 63(3):350-66.

566 37. Hirth A, Reybrouck T, Bjarnason-Wehrens B, Lawrenz W, Hoffmann A. Recommendations for participation in competitive and leisure sports in patients with congenital heart disease: a consensus document. Eur J Cardiovasc Prev Rehabil. 2006 Jun; 13(3):293-9. Available from: doi:10.1097/01.hjr.0000220574.22195.d6.

570 38. Takken T, Giardini A, Reybrouck T, Gewillig M, Hovels-Gurich HH, Longmuir PE, et al. Recommendations for physical activity, recreation sport, and exercise training in paediatric patients with congenital heart disease: a report from the Exercise, Basic & Translational Research Section of the European Association of Cardiovascular Prevention and Rehabilitation, the European Congenital Heart and Lung Exercise Group, and the Association for European Paediatric Cardiology. European Journal of Preventive Cardiology. 2012 Oct, 19(5),1034–1065. Available from: doi:10.1177/1741826711420000.

577 39. Longmuir PE, Brothers JA, de Ferranti SD, Hayman LL, Van Hare GF, Matherne GP, et al. American Heart Association Atherosclerosis, Hypertension and Obesity in Youth Committee of the Council on Cardiovascular Disease in the Young. Promotion of physical activity for children and adults with congenital heart disease: a scientific statement from the American Heart Association. Circulation. 2013 May 28, 127(21):2147-59. Available from: doi:10.1161/CIR.0b013e318293688f.

582 40. Schulz K, Altman D, Moher D. CONSORT 2010 statement: updated guidelines for reporting parallel group randomised trials. BMJ. 2010 Mar 23, 340(1):c332. Available from: doi:10.1136/bmj.c332.

585 41. Eldridge S, Lancaster G, Campbell M, Thabane L, Hopewell S, Coleman C, et al. Defining feasibility and pilot studies in preparation for randomised controlled trials: development of a conceptual framework. PLoS One. 2016; 11(3):e0150205. Available from: doi:10.1371/journal.pone.0150205.

589 42. Chan A, Tetzlaff J, Altman D, Laupacis A, Gøtzsche P, Krleža-Jerić K, et al. SPIRIT 2013 statement: defining standard protocol items for clinical trials. Ann Int Med. 2013, 158(3):200. Available from: doi:10.7326/0003-4819-158-3-201302050-00583.

For peer review only

43. Chan A, Tetzlaff J, Gotzsche P, Altman D, Mann H, Berlin J, et al. SPIRIT 2013 explanation and elaboration: guidance for protocols of clinical trials. BMJ. 2013 Jan 8, 346(15):e7586. Available from: doi:10.1136/bmj.e7586.

44. Sealed Envelope Ltd. Create a blocked randomisation list [online]. 2022 [cited 1 July 2023]. Available from https://www.sealedenvelope.com/simple-randomiser/v1/lists

45. Goodman R. The Strengths and Difficulties Questionnaire: A Research Note. Journal of Child Psychology and Psychiatry. 1997, 38:581-586. Available from doi:10.1111/j.1469-7610.1997.tb01545.x

46. Muris P, Meesters C, van den Berg F. The Strengths and Difficulties Questionnaire (SDQ). European Child & Adolescent Psychiatry. 2003, 12(1):1-8. Available from: doi:10.1007/s00787-003-0298-2

47. Varni JW, Seid M, Kurtin PS. PedsQL 4.0: Reliability and Validity of the PEdiatric Quality of Life Inventory Version 4.0 Generic Core Scales in Health and Patient Populations. Medical Care. 2001, 39(8):800-812.

48. Abda A, Bolduc ME, Tsimicalis A, Rennick J, Vatcher D, Brossard-Racine M. Psychosocial outcomes of children and adolescents with severe congenital heart defect: a systematic review and meta-analysis. J Pediatr Psychol. 2019, 44:463-477.

49. den Boer SL, Flipse DH, van der Meulen MH, Backx APCM, du Marchie Sarvaas GJ, Ten Harkel ADJ, et al. Six-Minute Walk Test as a Predictor for Outcome in Children with Dilated Cardiomyopathy and Chronic Stable Heart Failure. Pediatr Cardiol. 2017, 38(3):465-471. Available from: doi:10.1007/s00246-016-1536-y

50. Singh SJ, Morgan MD, Scott S, Walters D, Hardman AE. Development of a shuttle walking test of disability in patients with chronic airways obstruction. Thorax. 1992 Dec, 47(12):1019-24. Available from: doi:10.1136/thx.47.12.1019.

For peer review only

51. Cartwright-Hatton S, Mather A, Illingworth V, Brocki J, Harrington R, Wells A. Development and preliminary validation of the Meta-cognitions Questionnaire—Adolescent Version. Journal of Anxiety Disorders. 2004, 18(3):411-422. Available from: doi:10.1016/S0887-6185(02)00294-3

52. Myers S, Solem S, Wells A. The Metacognitions Questionnaire and Its Derivatives in Children and Adolescents: A Systematic Review of Psychometric Properties. Frontiers in Psychology. 2019 Sep 4, 10,1871. Available from: doi: 10.3389/fpsyg.2019.01871

53. Stevens K. Valuation of the Child Health Utility 9D Index. *PharmacoEconomics.* 2012, 30(8):729-747. Available from: doi:10.2165/11599120-000000000-00000

54. Lindvall K, Vaezghasemi M, Feldman I, Ivarsson A, Stevens K, Petersen S. Feasibility, reliability and validity of the health-related quality of life instrument Child Health Utility 9D (CHU9D) among school-aged children and adolescents in Sweden. Health and Quality of Life Outcomes. 2021, 19(1),193. Available from: doi:10.1186/s12955-021-01830-9

55. Lancaster GA, Dodd S, Williamson PR. Design and analysis of pilot studies: recommendations for good practice. J Eval Clin Pract. 2004, 10(2):307-312. Available from: doi:10.1111/j..2002.384.doc.x

56. Thijssen DH, Maiorana AJ, O'Driscoll G, Cable NT, Hopman MT, Green DJ. Impact of inactivity and exercise on the vasculature in humans. Eur J Apply Physiol. 2010, 108:845-875.

57. Malterud K, Siersma VD, Guassora AD. Sample Size in Qualitative Interview Studies: Guided by Information Power. Qualitative Health Research. 2016, 26(13):1753-1760. Available from: doi:10.1177/1049732315617444

58. Braun, V., & Clarke, V. Successful qualitative research: A practical guide for beginners. 2013. London: Sage.

59. Braun V, Clarke V. Using thematic analysis in psychology. Qual Res Psychol. 2006, 3(2):77–101.

60. Sekhon M, Cartwright M, Francis JJ. Acceptability of healthcare interventions: an overview of reviews and development of a theoretical framework. BMC Health Serv Res. 2017 Jan 26, 17(1),88. Available from: https://doi.org/10.1186/s12913-017-2031-8

For peer review only

61. Khan KA, Petrou S, Rivero-Arias O, Walters SJ, Boyle SE. Mapping EQ-5D utility scores from the PedsQL™ generic core scales. Pharmacoeconomics. 2014, 32(7),693-706.

62. Sweeney R, Chen G, Gold L, Mensah F, Wake M. Mapping PedsQL TM scores onto CHU9D utility scores: estimation, validation and a comparison of alternative instrument versions. Quality of Life Research. 2020, 29(3),639-652.

63. NHS England. New Review of Congenital Heart Services. 2013. Accessed From: https://www.england.nhs.uk/wp-content/uploads/2013/07/180713-item13.pdf

64. Shields GE, Wells A, Doherty P, Heagerty A, Buck D, Davies LM. Cost-effectiveness of cardiac rehabilitation: a systematic review. Heart. 2018, 104,1403–10. Available from: doi:10.1136/heartjnl-2017-312809

65. NHS England. Adult Congenital Heart Disease (ACHD) Specification. 2016. Accessed From: https://www.england.nhs.uk/wp-content/uploads/2018/08/Congenital-heart-disease-standards-and-specifications.pdf

66. Medical Research Council. Mental Health Research Goals 2020-2030. Available from: https://acmedsci.ac.uk/file-download/63608018

For peer review only

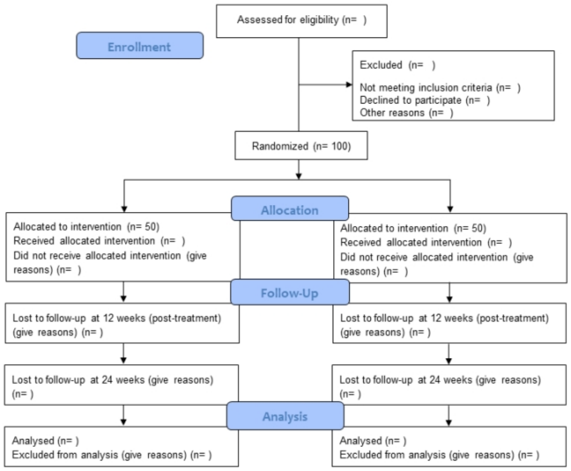

Figure 1. Trial Flow

338x190mm (96 x 96 DPI)

	STUDY PERIOD						
	Enrolment	Baseline	Allocation	Post-allocation (weeks)			
**TIMEPOINT**	Before starting CR			*1*	*10*	*12*	*24*
**ENROLMENT:**							
**Eligibility screen**	X						
**Informed consent**	X						
**Allocation**			X				
**INTERVENTIONS:**							
**Usual Care**				◆——	————	——◆	
**CR**				◆——	————	——◆	
**ASSESSMENTS:**							
Demographic Information Questionnaire (P)		X				X	X
SDQ (P)		X				X	X
PedsQol (CYP)		X				X	X
6MWT (CYP)		X				X	X
ISWT (CYP)		X				X	X
Physical Activity Monitoring (CYP)		X				X	X
MCQ-A (CYP)		X				X	X
CHU-9D (CYP)		X				X	X
SUQ (P)		X				X	X

Fig. 2 Schedule of enrolment, interventions and assessments. CR, Cardiac Rehabilitation; SDQ, Strength and Difficulties Questionnaire; PedsQol, Paediatric Quality of Life; 6MWT, Six minute walk test; ISWT, Incremental Shuttle Walk Test; MCQ-A, Metacognition Questionnaire-Adolescent; CHU-9D, Child Health Utility-9D; SUQ, Health and Social Care Service-Use Questionnaire; P, parent rated; CYP, child and young person rated

For peer review only

BMJ Open

Cardiac Rehabilitation for Children and Young People (CardioActive): Protocol for a Single-Blind Randomized Feasibility and Acceptability Study of a centre based cardiac rehabilitation programme versus usual care in 11-16-year-olds with heart conditions.

Journal:	*BMJ Open*
Manuscript ID	bmjopen-2023-077958.R1
Article Type:	Protocol
Date Submitted by the Author:	20-Nov-2023
Complete List of Authors:	Capobianco, Lora; Greater Manchester Mental Health NHS Foundation Trust,<br>Hann , Mark ; University of Manchester, Centre for Biostatistics and Manchester Academic Health Science Centre<br>McManus, Emma ; The University of Manchester, Health Organisation, Policy and Economics<br>Peters, Sarah; University of Manchester, Health Sciences<br>Doherty, Patrick Joseph; University of York, Health Science<br>Ciotti, Giovanna; Manchester University NHS Foundation Trust, Department of Pedeatric Cardiology<br>Murray, Joanne; Shrewsbury and Telford Hospital NHS Trust<br>Wells, Adrian; The University of Manchester, Division of Psychology and Mental Health; Greater Manchester Mental Health NHS Foundation Trust, Research and Innovation
**Primary Subject Heading**:	Cardiovascular medicine
Secondary Subject Heading:	Mental health, Paediatrics, Rehabilitation medicine
Keywords:	MENTAL HEALTH, Paediatric cardiology < CARDIOLOGY, REHABILITATION MEDICINE

SCHOLARONE™
Manuscripts

Cardiac Rehabilitation for Children and Young People (CardioActive): Protocol for a Single-Blind Randomized Feasibility and Acceptability Study of a centre based cardiac rehabilitation programme versus usual care in 11-16-year-olds with heart conditions.

Lora Capobianco[1,2], Mark Hann[3], Emma McManus[4], Sarah Peters[2,5,], Patrick Doherty[6], Giovanna Ciotti[7], Joanne Murray[8], Adrian Wells[1,2]

[1] Division of Psychology and Mental Health, School of Psychological Sciences, Faculty of Biology, Medicine and Health, The University of Manchester, Manchester, UK.

[2]Research and Innovation, Greater Manchester Mental Health NHS Foundation Trust, Manchester, UK

[3]Division of Population Health, Health Services Research, and Primary Care, Biostatistics Group, Faculty of Biology, Medicine, and Health, The University of Manchester, Manchester, UK.

[4]Health Organisation, Policy and Economics (HOPE), Division of Population Health, Health Services Research and Primary Care, School of Health Services, Faculty of Biology, Medicine and Health, The University of Manchester, Manchester, UK.

[5]Manchester Centre for Health Psychology, Faculty of Biology, Medicine and Health, Division of Psychology and Mental Health, University of Manchester

[6] Department of Health Sciences, University of York, York, UK

[7] Department of Pedeatric Cardiology, Manchester University NHS Foundation Trust, Manchester, UK

[8] Psychology Service, Shrewsbury and Telford Hospital NHS Trust, Shrewsbury, UK

Corresponding Author: Dr. Lora Capobianco, lora.capobianco@gmmh.nhs.uk

For peer review only

Abstract

Background: Congenital heart conditions are amongst the most common non-communicable diseases in children and young people (CYP), affecting 13.9 million CYP globally. While survival rates are increasing, support for young people adjusting to life with a heart condition is lacking. Furthermore, 1 in 3 CYP with heart conditions also experience anxiety, depression, or adjustment disorder, for which little support is offered. While adults are offered cardiac rehabilitation (CR) to support their mental and physical health, this is not offered for CYP.

One way to overcome this is to evaluate a CR programme comprising exercise with mental health support (CardioActive; CA) for CYP with heart conditions. The exercise and mental health components are informed by the metacognitive model, which has shown to be effective in treating anxiety and depression in CYP and associated with improving psychological outcomes in adult CR.

Method and Analysis: The study is a single blind parallel randomized feasibility trial comparing a CR programme (CA) plus usual care against usual care alone with 100 CYP (50 per arm) aged 11-16 diagnosed with a heart condition. CA will include six group exercise, lifestyle and mental health modules. Usual care consists of routine outpatient management. Participants will be assessed at three time points: baseline, 3-month (post-treatment) and 6-month follow up. Primary outcomes are feasibility and acceptability (i.e., referral rates, recruitment and retention rates, attendance at the intervention, rate of return and level of completion of follow up data). Co-primary symptom outcomes (strength and difficulties questionnaire and paediatric quality of life), and a range of secondary outcomes will be administered at each time point. A nested qualitative study will investigate CYP, parents, and healthcare staff views of CR and its components, and staff's experience of delivering CA. Preliminary health economic data will be collected to inform future cost-effectiveness analyses. Descriptive data on study processes and clinical outcomes will be reported. Data analysis will follow intention to treat. Qualitative data will be analysed using thematic analysis and the theoretical framework of acceptability.

For peer review only

Ethics and Dissemination: Ethical approval was granted on 14/02/23 by the Greater Manchester East Research Ethics Committee (: 22/NW/0367). The results will be disseminated through peer reviewed journals, conference presentations, and local dissemination.

Trial registration: ISRCTN50031147; NCT05968521

Keywords: cardiac, youth, mental health, metacognition, metacognitive therapy, rehabilitation

Strengths and Limitations of This Study

- The study will evaluate anovel cardiac rehabilitation intervention (CardioActive; CA) for children and young people with heart conditions which includes both mental and physical health support based on evidenced based psychological theory.
- As the study is feasibility, the sample size is limited and the study is not powered to determine efficacy.
- Data collection will be conducted by researchers blind to participant allocation

Introduction

Congenital heart disease (CHD) affects 13.8 million children and young people (CYP) under the age of 20 globally [1]. Survivors of CHD face challenges and comorbidities which impact their ability to function [2], future employment, and progression into independent adulthood [3, 4]. CYP with heart conditions have a reduced quality of life and poorer psychological functioning [5-7, as 41% of CYP with CHD experience significant difficulties adjusting to their heart condition, and 30% experience clinically significant anxiety or depression [7-13]. In addition, overprotection from parents, educators, and healthcare staff contribute to reduced physical activity, and exercise intolerance [14-17]. This is particularly important as sedentary lifestyles are a risk factor for atherosclerosis, cardiovascular disease, obesity and diabetes [18].

A possible means of improving physical and mental health outcomes would be to facilitate access to a cardiac rehabilitation (CR) programme that improves healthy behaviours such as exercise and

For peer review only

For peer review only - http://bmjopen.bmj.com/site/about/guidelines.xhtml

psychological self-management skills. CR programmes provide a unique opportunity to improve physical health, provide education around lifestyle and risk factors, and provide psychological support [19,20]. In the UK, following a cardiac event, adults are encouraged to attend CR programmes, which have been recommended by the National Health Service (21), the Department of Health, National Institute for Health and Care Excellence (22) guidelines (CG172, CG94 and NG106) and the British Association for Cardiovascular Prevention and Rehabilitation (23 In adults CR programmes have been found to reduce patient mortality, morbidity, hospital re-admissions, improve quality of life, and be a cost-effective intervention [24, 25]. While CR programs are accessible for adults, equivalent programs are not routinely available for CYP living with heart conditions.

Previous systematic reviews and meta-analyses of paediatric CR have indicated that paediatric CR programmes improve physical, developmental, cognitive, and psychosocial outcomes [24, 26-28]. Akamagwuna and Badaly [24] conducted a systematic review of 20 paediatric cardiac rehabilitation programmes. They found that both home-based and outpatient programmes were associated with improvements in cardiorespiratory outcomes, quality of life and psychosocial functioning. Previous youth CR programmes have predominantly focused on improving physical health outcomes, with less attention given to mental health components, despite 41% of patients struggling with adjusting to living with a heart condition. Tesson et al [29] conducted a systematic review of psychological interventions for childhood-onset heart disease and found only two studies focused on interventions for CYP [30,31]. However, the results were disappointing, as they did not lower anxiety, depression [31] or improve quality of life [30]. CR with CYP could benefit from routine exercise programmes combined with psychological input aimed at improving adjustment and mental health outcomes.

Drawing on the literature on physical and psychological effects of CR techniques in CYP [29-32] and informed by theory and research on metacognitive therapy in adult CR and mental health [33-35]), we have devised CardioActive (CA), a 6-session CR package for use with CYP. The duration of the CA

For peer review only

97 programme is briefer than previous programmes wherepatients have on average received 2.5 sessions per week for 16 weeks [24, 26-28, 32]. The physical health component has been informed by recommendations for physical activity in CYP with congenital heart disease (CHD;36-39), and the latest systematic reviews and meta-analyses which suggest that programmes should include a combination of endurance and strength-based exercises (29-32). The mental health/adjustment component of CA is informed by the metacognitive model [40-42] of anxiety, depression and stress and focuses on helping CYP to reduce their worry and unhelpful self-monitoring processes. According to the metacognitive model psychological distress and failure to adjust is maintained by a style of thinking termed the cognitive attentional syndrome (CAS). The CAS is characterized by repetitive negative thinking (worry, dwelling), monitoring for threat (i.e., checking for symptoms), inflexible attention, and engaging with unhelpful coping strategies (i.e., avoiding exercise, resting more, poor eating, avoidance). Metacognitive approaches may be more appropriate than cognitive therapy approaches as cognitive therapy utilizes techniques such as reality testing patient's thoughts, which in heart disease patients are often realistic (i.e. "what if my heart disease gets worse?") and may not be amenable to cognitive therapy techniques [43]. This approach has been usefully applied in adult CR to improve psychological outcomes [34].

While the metacognitive approach is promising, further research is needed to understand the views and responses to the metacognitive approach delivered as part of CR for CYP. As such, the aim of the proposed study is to develop a cardiac rehabilitation programme (CardioActive; CA) which incorporates mental health support for CYP and to evaluate the acceptability and feasibility of the programme in comparison to usual care.

Methods

Design

The current study is a parallel single-blind randomised feasibility study in children and young people aged 11-16 with a heart condition with 12 and 24 weeks follow up, comparing routine clinical

For peer review only - http://bmjopen.bmj.com/site/about/guidelines.xhtml

management (treatment as usual (TAU)) against the CardioActive (CA) programme. Qualitative investigation will be embedded within the trial. Preliminary economic data will be obtained to inform the optimum way of evaluating cost-effectiveness in any subsequent definitive trial. See Figure 1 for an overview of the trial design according to Consolidated Standards of Reporting Trials (CONSORT) guidelines [44] [45]. The recommendations for the Interventional Trials 2013 Standard Protocol Items: Recommendations for Interventional Trials; SPIRIT) Checklist [46] [47] is included in Additional file 1. Figure 2 outlines the schedule of recruitment, interventions, and assessment.

INSERT FIGURE 1 HERE

INSERT FIGURE 2 HERE

Trial population

The trial population are children and adolescents attending cardiology outpatient services.

Eligibility Criteria

In order to be eligible to take part in the study participants will meet the following inclusion and exclusion criteria:

Inclusion Criteria

1. Aged between 11-16 years.
2. Ability to consent to participate.
3. Native fluency in English language.
4. Diagnosed with at least one of the following: congenital heart disease (all subtypes), cardiomyopathy, cardiac arrhythmia, heart failure, post-cerebrovascular event, post-heart valve repair/replacement.

For peer review only

For peer review only - http://bmjopen.bmj.com/site/about/guidelines.xhtml

Participants will be excluded if they meet one or more of the following:

1. Presence of significant risk or safeguarding concerns (i.e., exercise risk)
2. Head injury/organic impairment
3. Significant communication and/or social difficulties

Only patients with a formal diagnosis or under assessment for one of the exclusion criteria will be excluded from the study.

Recruitment and allocation

Participants will be screened for eligibility during routine clinic appointments by clinicians and/or cardiac team members. Clinicians/cardiac team members will ask eligible patients and their parent/caregiver if they are interested in hearing more about the research study and if their contact details can be passed onto the research team. The research team will then contact eligible and interested patients and their caregivers to provide further information about the study, answer any questions, arrange for consent to be obtained, and collect baseline data. Once baseline data have been collected, participants will be randomised to a study arm using sealed-envelope software [48]. Randomisation (at the individual level) will be independent and concealed, after stratification by age (11-13; 14-16) and sex (male; female). Within strata, allocation will occur within blocks of size 4 or 6, determined at random and with equal probability by the software. This will minimise future allocation 'predictability'. A member of the research team not blinded to allocation will contact parents/caregivers to inform them of the allocation within 2 days. Blinding of allocation will be maintained for quantitative research assistants, and the trial statistician until all outcome measures for all subjects have been collected. The Trial Executive Committee (TEC) will regularly monitor any unblinding's and corrective action will be implemented if needed.

For peer review only

For peer review only - http://bmjopen.bmj.com/site/about/guidelines.xhtml

169 **Trial Conditions**

170 **CardioActive Programme**

171 The programme will consist of six face to face group-based sessions lasting up to 90 minutes
172 of a structured exercise programme with integrated educational and lifestyle modules, and a
173 psychological component delivered at the recruiting hospital sites. Exercise sessions have been
174 reviewed by a physiotherapist with experience working with patients with heart conditions and a
175 child cardiologist who have deemed the exercise safe for CYP to engage in. Patients are free to
176 stop the exercise if they begin to feel unwell. Lifestyle modules will focus on modifiable risk
177 factors. The psychological treatment component (Metacognitive Therapy; MCT) is integrated
178 throughout each session and focusses on controlling worry and using adaptive attention
179 strategies. Sessions include group discussions, experiential learning and homework tasks that
180 participants are asked to complete between sessions. Participants in this treatment arm will also
181 receive routine clinical outpatient management (treatment as usual) alongside CR.

182 **Treatment as Usual (TAU)**

183 TAU will consist of routine clinical outpatient management, routine screening, and
184 medication management, which includes a review with their cardiologist or cardiac specialist
185 nurse every 6 to 12 months, depending on the nature and severity of the heart condition.

186 **Staff training and supervision**

187 The intervention will be co-delivered by two cardiology staff members. Staff from the
188 cardiology department will receive two full training days in the intervention followed by six
189 weeks of supervised practise in delivering the intervention to a pilot group of volunteers. A
190 further workshop will address any challenges the staff's experienced when delivering the
191 intervention. Staff's delivery of the intervention will be monitored by listening to audio-
192 recordings of sessions and they will receive supervision throughout the trial. Adherence to the

For peer review only

For peer review only - http://bmjopen.bmj.com/site/about/guidelines.xhtml

intervention will be assessed using an adherence checklist completed by the staff at each session.

Data collection

The planned study recruitment date is March 2024, with the end of follow-up marking study completion in June 2025. There will be three follow-up assessment points: baseline, 12 weeks (end of treatment), and 24 weeks (follow-up). To ensure completeness of the questionnaires, participants will be asked to arrange a visit, either at an National Health Service (NHS) site or in their own home, at each timepoint. Participants total time involvement in the study will be 24 weeks. Participants will be reimbursed with a £10 shopping voucher for completing each assessment.

Criteria for discontinuation

Participants may withdraw from the study at any time, without any consequences to themselves, their health care, or their ability to take part in future research. Participants who withdraw will continue to receive usual care. Participants can also be withdrawn at the request of the Chief Investigator or cardiology service, but this would only happen if a participant's life or long-term health or welfare was at risk from continued participation in the study.

Outcomes

The primary outcomes for the study are feasibility and acceptability outcomes. Feasibility will be assessed using referral rates, recruitment (i.e., number of patients consented and randomized, number of patients declined the study) and retention rates (i.e., number of patients withdrawn from the study, lost to follow up), and attendance at the intervention. Qualitative semi-structured focus groups or interviews, as preferred, will be used to understand children and young people, caregivers, and healthcare professionals' views of acceptability of the intervention and targets for improvement. All outcomes listed below will be obtained at each assessment time point.

For peer review only

For peer review only - http://bmjopen.bmj.com/site/about/guidelines.xhtml

Measures*Strength and Difficulties Questionnaire (SDQ; [49]).*

The SDQ is a behavioural measure completed by the caregiver and child for children aged 2 to 17, across five subscales: emotion, hyperactivity, conduct, peer relations and pro-social behaviour. Each subscale contains five items, and each item is scored "Not True", "Somewhat True" or "Completely True". Scores for the subscales range from 0-10, with total difficulties score (0 – 40) generated by summing together the scores from all subscales except pro-social. Higher scores on the pro-social scale reflect strengths whereas higher scores across other subscales reflect difficulties [49]. The SDQ subscales have satisfactory internal consistency (mean Cronbach's alpha = 0.70) and test-retest reliability ranging from 0.75 – 0.91 [50].

For peer review only

Paediatric Quality of Life (PedsQol: [51]).

The PedsQol consists of 23 items which measures health related quality of life including physical, emotional, social, and school functioning, and is commonly used in paediatric cardiology [5, 52]. The items are scored on a Likert scale ranging from 0 (Never) to 4 (Almost always). Total scores range from 0-100, with higher scores indicating better quality of life. The PedsQol will be completed by CYP.*Six minute walk test (6MWT: [53]).*

The 6MWT is used to assess aerobic capacity and endurance. Participants are asked to walk as far as possible for six minutes and the total distance covered during this time is used as the outcome. Performance on the 6MWT has been predictive of morbidity and mortality whereby poorer performance is associated with increased mortality [53].

Incremental Shuttle Walk Test (ISWT: [54])

The ISWT is used to assess exercise capacity. The ISWT is a 12-level test (1 min in each level), whereby participants walk up and down a 10 m course with increasing acceleration of 0.17 m/s up to a maximum speed of 2.37 m/s. The walking speed is dictated by an audio signal. The test ends when the subject has a heart rate greater than 85% of their predicted maximum, is limited by dyspnoea, or

For peer review only - http://bmjopen.bmj.com/site/about/guidelines.xhtml

when the subject is unable to maintain the required speed and does not complete a shuttle for a second consecutive time. The distance covered from the number of shuttles will be calculated.

Physical Activity Monitoring.

Participants will wear an accelerometer (Actigraph) for five days to monitor physical activity levels at each assessment. Data collected via the Actigraph includes total movement, moderate to vigorous physical activity, non-sedentary time, step count, and energy expenditure.

Metacognition Questionnaire-Adolescent (MCQ-A; [55]).

The MCQ-A assesses metacognitive beliefs (beliefs about thinking) across five subscales: uncontrollability and dangerousness of worry, need to control, cognitive self-consciousness, positive beliefs about worry, and cognitive confidence. The 30 items are scored on a Likert scale from 1 (do not agree) to 4 (agree very much) with total scores ranging from 30-120 and 6-30 for each subscale [55]. The internal consistency of the MCQ-A across total scores and most subscales have been supported with adequate to excellent Cronbach alphas (0.76 – 0.92) [56]. This MCQ-A will be completed by CYP.

Child Health Utility-9D (CHU-9D: [57])

The CHU-9D measures paediatric quality of life using nine dimensions (including worried, sad, pain, tired, annoyed, schoolwork/homework, sleep, daily routine, and ability to join in activities) and is suitable for CYP aged between 7–17-years-old. Each item has 5 levels ranging from no problems to inability to do the item [57]. The CHU-9D has strong internal consistency (Cronbach's alpha = 0.79) and strong test-retest reliability (Interclass correlation coefficients = 0.75) [58]. The CHU-9D will be completed by CYP.

Demographic information questionnaire

For peer review only

The demographic questionnaire will collect variables including child's age, sex, weight, height, body mass index (BMI), type of heart problem, comorbid mental and physical illnesses, ethnicity, medication, socioeconomic status, parental occupational status. This will be completed by parents/primary caregivers.

Health and Social Care Service-Use Questionnaire (SUQ)

The SUQ will include questions about whether the child has used any primary, secondary, or community-based health and social care and how often they used the service in the last 16 weeks (baseline study visit) or since the last assessment (follow-up study visits). The questionnaire will be developed from existing child relevant service use questionnaires held by the co-applicants and through discussion with the patient and public involvement (PPI) representative, parent advisory group and clinical members of the study team. This will be completed by parents/primary caregivers.

Qualitative Nested Study

We will investigate patients', caregivers', and healthcare professionals' experiences of the CardioActive programme, targets for improvement, perceived acceptability of components of CR, and examine participants experience of living with/managing heart conditions using semi-structured interviews with CYP (n = 20), caregivers (n = 10), and healthcare professionals (n = 10). Sampling will be purposive to ensure variation in key variables including age, type of health condition, relationship to CYP, and discipline of health care professional (e.g., physiotherapist, cardiologist, clinical psychologist etc). We will endeavour to recruit patients with a range of experiences of CR including those for whom engagement has been low as well as those who have attended regularly. We will explore the acceptability of CR, factors influencing participants' engagement, and barriers and enablers to CR being implemented within services. We will also explore with participants their experiences of key trial processes (e.g., outcome measures, recruitment, and randomisation procedures) to inform the design of a definitive trial. An

For peer review only - http://bmjopen.bmj.com/site/about/guidelines.xhtml

interview topic guide will be used in order to maintain a consistent structure. Members from the PPI group will be involved in the development of the interview schedule and piloting for the qualitative interviews. Participants will be reimbursed for taking part in qualitative interviews or focus groups.

Sample size calculation

Feasibility trials are not powered to provide a definitive effectiveness analysis. Therefore, the sample size is based on having sufficient patients to evaluate the acceptability/feasibility of the intervention (as measured by patient acceptability and adherence ratings, recruitment, and retention rates), and to obtain a provisional estimate of the 'promise' of the intervention (e.g. an 80% C.I.) for powering a future definitive randomised controlled trial (RCT). We will recruit 50 patients per arm (total N = 100), which would allow for an overall attrition rate of up to 20% to be determined to within eight absolute percentage points with 95% confidence. The total sample size is in line with recommendations by Lancaster et al [59]. This will also be sufficient to estimate key 'clinical' parameters, such as the standard deviation of potential outcomes (in the larger trial), with adequate precision, for which samples of 40 are generally sufficient [60].

Our qualitative sample has been informed by prior experience and published guidance [61]. Previous research suggests that a sample size of 12 can achieve data saturation [62]. The exact number will be determined by data sufficiency, achieving adequate variance in recruitment and thematic saturation, however we are confident this estimate will be sufficient to answer the relevant research questions.

Analyses

Quantitative analyses

As this is a feasibility study, we will not be carrying out hypothesis testing to determine if the intervention is effective. Instead, descriptive data on study processes and clinical outcomes will be

For peer review only

For peer review only - http://bmjopen.bmj.com/site/about/guidelines.xhtml

reported. Following an Intention-To-Treat (ITT) protocol, we will present data relating to participant recruitment, randomisation, retention at follow up and drop out rate in a CONSORT flow chart. The attrition rate will be calculated (with a 95% confidence interval) and reasons for drop-out will be recorded where possible. We will also assess rates of missing data (including which elements) on specific questionnaires and whether any of the measures display floor and/ or ceiling effects.

We will summarise by trial arm, as appropriate (e.g., mean/ standard deviation (SD); median/ inter-quartile range; proportion/95% confidence interval; data range), data for all potential (i.e. for the larger trial) clinical primary outcome measures. We will also report 80% confidence intervals for the trial arm co-efficient from appropriate regression analyses (adjusting for age, gender and the corresponding baseline outcome) by way of investigating the promise of the CardioActive program. This information, the SD of the SDQ and the PedsQoL, the estimated attrition rate and recruitment-based data will be used to help inform the sample size calculation for the large-scale, definitive RCT (in addition to other (published) sources). The statistical analysis plan will be produced by MH (statistician) prior to the examination of outcomes.

Qualitative analyses

All focus groups and interviews will be transcribed verbatim. Thematic Analysis (TA) [63] and the theoretical framework of acceptability [64] will be used, allowing for both inductive and deductive coding. Members from the PPIE group will provide feedback on emerging analyses.

Economic analyses

We will evaluate the feasibility of conducting an economic evaluation of CR, by exploring the costs and outcomes associated with CR compared to standard care. We will co-design a health-resource use questionnaire with our PPIE group to capture any changes in healthcare resource use during the study, which will be completed by CYP and their guardians. We will explore resource use from the perspective of the NHS and personal social services, as well as taking a wider perspective to

For peer review only

For peer review only - http://bmjopen.bmj.com/site/about/guidelines.xhtml

337 examine if there are any patient out-of-pocket expenses. We will also examine the resources and
338 their associated costs required to develop and deliver the intervention.

339 We will measure health outcomes using two measures, as described above: the CHU-9D and
340 PedsQol [65]. The CHU-9D has a published value set in which to generate utility scores [57] and
341 therefore quality adjusted life years. There is also a published mapping algorithm to transform the
342 PedsQol to the EQ-5D [65] [66]. We will examine the acceptability of both measures to the CYP and
343 assess data completeness, to inform which questionnaire is most appropriate for use in the
344 definitive study, or if indeed, another measure of paediatric quality of life would be more
345 appropriate.

346 We will conduct a preliminary economic evaluation using the data collected, to determine
347 whether CR plus TAU would be cost-effective from the perspective of the NHS and Personal Social
348 Services.

349 Multiple imputation and censored data analysis techniques will be used to separately
350 impute missing observations from participants who complete follow-up, and missing follow-up data
351 for participants lost to follow-up. The primary and sensitivity economic analyses will be controlled
352 for key baseline covariates or characteristics. Cost effectiveness acceptability curves will assess the
353 likely cost effectiveness of the intervention and uncertainty in the observed data. This cost-
354 effectiveness analysis will be limited due to the sample size and the overall health economic work
355 will focus on informing a definitive trial.

356 **Trial management and oversight arrangement**

357 The trial is managed by a Trial Executive Committee (TEC) which includes those individuals
358 responsible for the day-to-day management of the study including the chief investigator, co-
359 investigators and identified collaborators, principal investigators, and the study statistician.
360 Notwithstanding the legal obligations of the Sponsor and Chief Investigator, the TEC has operational

For peer review only

responsibility for the conduct of the study including monitoring overall progress to ensure the protocol is adhered to and to take appropriate action to safeguard the patients and the quality of the study. There will also be patient and public involvement groups (adult and CYP) which will meet at least quarterly and provide advice and feedback on a range of trial-related activities, e.g., reviewing study documents. The end of the study will be reported to the REC within the required timeframe if the study is terminated prematurely. Investigators will inform patients of any premature termination of the study and ensure that the appropriate follow up is arranged for all involved. Following the end of the study a summary report of the study will be provided to the REC within the required timeframe.

Data management

Participants will be allocated a study identity code number for use on all study documents and the electronic database. The study team will make a separate confidential database for the participant's name, date of birth and study identity code to permit identification of participants enrolled in the study e.g., for follow-up. All other information will be anonymised. All study documents (including participant's written consent forms) which are to be held at the participating centres will be held in strictest confidence. Access to patient information shall be restricted to authorized persons. Data will only be available through restricted, shared areas on the secure Greater Manchester Mental Health (GMMH) and University of Manchester computer systems (password and username secured). Baseline and follow-up data, which is anonymous data, will be stored in locked filing cabinets at GMMH. These data will be entered into an electronic database for analysis purposes by study team members blind to trial arm allocation. All computers are password protected and adhere to the secure storage policies of the NHS trust and University of Manchester.

Safety reporting

Adverse events (AEs) and serious adverse events (SAEs) will be monitored throughout the study. AEs are defined as any untoward medical occurrence in a patient or study-specific

For peer review only

intervention and which does not necessarily have a causal relationship with the intervention (i.e., deterioration in cardiac health not associated with the intervention). SAEs are defined as AEs that result in death, are life-threatening or require hospitalisation. Adverse reactions (ARs) are an untoward medical or unintendical medical response in a patient to a study specific intervention which has a causal relationship with the intervention (i.e., physical injury due to the intervention). Serious adverse reactions (SARs) are defined as ARs that result in death, are life-threatening or require hospitalisation. All AEs, SAEs, ARs and SARs will be reviewed by the trial's child clinical psychologist and paediatric consultant cardiologist within 24 hours of reporting. Any adverse events identified as likely to be caused by the intervention during the period of recruitment or intervention on the study will be recorded at the study site using an AE/SAE record form which will be completed by the health professionals. Study Research Assistant (RA) and study Principal Investigators (PIs) will be informed, and information inputted onto a secure database. The CIs will also assess if the nature of the event is likely to have been induced because of the study intervention or processes. Any that are deemed be serious and related to the intervention will be reported to the Sponsor, the study executive committee, and ethics committee within 24 hours of the event.

Ethics and Dissemination

The study received full ethical approval on February 14, 2023, from North West – Greater Manchester East Research Ethics Committee, Research Ethics Committee (REC) REC ref: 22/NW/0367, IRAS ID 319134. The trial has been registered with clinicatials.gov (NCT05968521) and with ISRCTN (ISRCTN50031147). The study protocol used was Version 6: 21/06/2023. Any modifications to the trial will be submitted for further ethical approval and approved changes will be documented and communicated to the REC, trial registry, executive committee, and all relevant parties. The study will be conducted in accordance with the ethical principles that have their origin in the Declaration of Helsinki (1996), the principles of Good Clinical Practice, and the UK Policy Framework for Health and Social Care Research (2017). GMMH acts as the sponsor for this study. As

For peer review only - http://bmjopen.bmj.com/site/about/guidelines.xhtml

411 the sponsor is an NHS organisation, the NHS indemnity scheme will apply. Participating sites will be liable for clinical negligence and other negligent harm to participants taking part in the study and covered by the duty of care owed to them by the sites concerned.

Written informed consent will be obtained from all participants and their parents/caregivers. Participants will be free to withdraw from the study at any time without providing a reason or their care being impacted. All the information collected during this trial will be confidential and held in accordance with NHS Data Protection guidelines and Good Clinical Practice guidelines. Confidentiality will only be breached if participants disclose information which may indicate that there is a risk of harm to themselves or others. Every opportunity to discuss any possible breaches of confidentiality with participants will be taken prior to informing any appropriate agencies, e.g., Children and Adolescent Mental Health Services (CAMHS), GP or A&E services.

All researchers and study site staff involved with the study must comply with the requirements of the Data Protection Act 1998 regarding the collection, storage, processing, and disclosure of personal information, and will uphold the Act's core principles. Audio-recordings and transcriptions of interviews will be stored on NHS drives, which are password protected and designed for the storage of confidential research material. Interviews which are transcribed will be anonymised at the point of transcription. Any third party involved with transcribing of interviews will sign a confidentiality agreement and be fully instructed in how to anonymise transcripts.

The main study results will be published in the name of the study in a peer-reviewed journal, on behalf of all collaborators. The manuscript will be prepared by a writing group, appointed from amongst the trial executive committee (TEC) . All participating centres and clinicians will be acknowledged in this publication. All presentations and publications relating to the study must be authorised by the study executive committee and sponsor, on whose behalf publications should usually be made. Authorship of any secondary publications resulting from the intervention will

For peer review only - http://bmjopen.bmj.com/site/about/guidelines.xhtml

reflect the intellectual and time input into these studies and will not be the same as on the primary publication. No investigator may present or attempt to publish data relating to this study without prior permission from the TEC . The findings will also be presented at national, international, and regional conferences and in public involvement events where the information from this study is relevant.

Patient and Public Involvement

A youth PPI group comprised of young people with lived experience of a heart condition (with and without mental health difficulties) has been created along with an adult PPI group comprised of parents, caregivers, and adult heart condition patients (with and without mental health difficulties). Both PPI groups have aided in the development of the CardioActive programme, in obtaining ethical approval, co-developed patient facing materials, helped to develop the patient recruitment and retention plan, have helped to develop the study name and logo, and will further assist with retention, development of the dissemination plan, and with dissemination of research findings.

Discussion

CHD presents as one of the most common non-communicable childhood diseases. While medical advances have improved survival rates, young people with heart conditions continue to face significant challenges, comorbidities, reduced quality of life and increased psychological distress. NHS England has highlighted that cardiac services must be provided in a way that allows the best outcomes for all patients, not only in reducing mortality, but also in decreasing disability and improving opportunities to lead better lives [67].

A CR programme that integrates mental and physical healthcare has the potential to meet NHS objectives [68] which aims to ensure that CYP, their family and carers have an understanding of the

For peer review only

For peer review only - http://bmjopen.bmj.com/site/about/guidelines.xhtml

patient's condition, its impact on their health and future, and an understanding of the condition's psychological, social, and cultural factors in addition to service specifications for cardiac disease [69].

The current study is limited by the sample size, and it is not powered to evaluate efficacy of the intervention. Despite this, the study will attempt to provide data on important logistical and clinical outcomes, which will help inform the power calculation for a full trial.

Despite the limitations, the present study would meet service standards and fits within current priorities for closer integration of psychological and physical health services [19,20,70]. Development and evaluation of a CR programme is an important platform for the progression of young person's cardiac services and will provide valuable qualitative and quantitative data to support the design of a future large-scale definitive trial to test the effectiveness of CardioActive for CYP with CHD.

Figures and Captions

Figure 1. Trial Flow Diagram

Figure 2. Schedule of Events

Author Contributions

LC is the chief investigator and conceived the study, designed, and developed the initial trial protocol, developed the trial intervention, and contributed to the first and subsequent drafts of the manuscript. AW is the co-chief investigator and contributed to the study conception, designed, and developed the initial trial protocol, developed the trial intervention, and contributed to the first and subsequent drafts of the manuscript. AW provided translational guidance in applying the metacognitive model in CR. MH contributed to the study design, statistical analysis, and sample size and wrote the quantitative evaluation section of the manuscript. EM developed the health economics components of the protocol and wrote these sections of the manuscript. GC contributed

For peer review only

to the development of the trial recruitment strategy. PD contributed to study design. JM contributed to adverse event reporting. SP developed the qualitative components of the protocol and wrote these sections of the manuscript. All authors edited the manuscript and read and approved the final version.

Consent for publication

Not applicable.

Availability of data and materials

The datasets generated and/or analysed during the current study are not publicly available due to ongoing data collection but are available from the corresponding author on reasonable request on completion of data collection and data analysis.

Competing interests

AW is the creator of Metacognitive Therapy, and co-director of the MCT Institute.

Funding

The study is funded by the National Institute for Health Research (NIHR) under its Research for Patient Benefit Grant (Grant Reference Number: NIHR203634). The views expressed are those of the author(s) and not necessarily those of the NIHR or Department of Health.

For peer review only

References

1. Children & Non-Communicable Disease: Global Burden Report 2019 – NCD Child. (2019). Retrieved 4 May 2022, from https://www.ncdchild.org/2019/01/28/children-non-communicable-disease-global-burden-report-2019/
2. Cooper DS, Jacobs JP, Chai PJ, Jaggers J, Barach P, Beekman RH, et al. Pulmonary complications associated with the treatment of patients with congenital cardiac disease: consensus definitions from the multi-societal database committee for pediatric and congenital heart disease. Cardiol Young. 2008; 18 Suppl 2:215–221.
3. M. Kamphuis, T. Vogels, J. Ottenkamp, E.E. van der Wall, S.P. Verloove-Vanhorick, H.W. Vliegen. Employment in adults with congenital heart disease. Arch Pediatr Adolesc Med. 2002; 156(11):1143-1148
4. Brandhagen DJ, Feldt RH, Williams DE. Long-term psychologic implications of congenital heart disease: a 25-year follow-up. Mayo Clin Proc. 1991; 66(5):474-479
5. Vigl M, Niggemeyer E, Hager A,Schwedler G, Kropf S, Bauer U. The importance of socio-demographic factors for the quality of life of adults with congenital heart disease. Qual Life Res Int J Qual Life Asp Treat Care Rehab. 2011; 20(2):169-177.
6. Moons P, Van Deyk K, Marquet K, De Bleser L, De Geest S, Budts W. Profile of adults with congenital heart disease having a good, moderate, or poor quality of life: a cluster analytic study. Eur J Cardiovasc Nurs. 2009; 8(2):151-157.
7. Latal, S. Helfricht, J.E. Fischer, U. Bauersfeld, M.A. Landolt. Psychological adjustment and quality of life in children and adolescents following open-heart surgery for congenital heart disease: a systematic review. BMC Pediatr. 2009; 9(6).
8. Abda A, Bolduc ME, Tsimicalis A, Rennick J, Vatcher D, Brossard-Racine M. Psychosocial outcomes of children and adolescents with severe congenital heart defect: a systematic review and meta-analysis. J Pediatr Psychol. 2019; 44:463-477.

For peer review only

For peer review only - http://bmjopen.bmj.com/site/about/guidelines.xhtml

9. Wang QF, Hay M, Clarke D, Menahem S. The Prevalence and Predictors of Anxiety and Depression in Adolescents with Heart Disease. Journal of Pediatrics. 2012; 161(5):943-946.

10. Awaad MI, Darahim KE. Depression and anxiety in adolescents with congenital heart disease. Middle East Current Psychiatry. 2015; 22,2-8.

11. Gonzalez, V.J., Kimbro, R.T., Cutitta, K.E., Shakosky, J.C., Bilal, M.F, Penny DJ, Lopez KN. Mental Health Disorders in Children With Congenital Heart Disease. Pediatrics February. 2021; 147(2):e20201693. doi: 10.1542/peds.2020-1693

12. Anton CM, Drake MB, Butts RJ, Cao T, Rezaeizadeh A, Trivedi MH, et al. Electronic Mental Health Screening in a Pediatric Heart Failure and Transplant Clinic. J Clin Psychol Med Settings. 2021; 28(4):815-825. doi:10.1007/s10880-021-09770-8

13. Longmuir PE, Sampson M, Ham J, Weekes M, Patel BJ, Gow RM. The mental health of adolescents and pre-adolescents living with inherited arrhythmia syndromes: a systematic review of the literature. Cardiol Young. 2018; 28(5):621-631. doi:10.1017/S104795111700289X

14. Belardinelli R, Georgiou D, Cianci G, A. Purcaro A. Randomized, controlled trial of long-term moderate exercise training in chronic heart failure: effects on functional capacity, quality of life, and clinical outcome. Circulation. 1999; 99(9):1173-1182

15. Casey FA, Stewart M, McCusker CG, Morrison ML, Molloy B, Doherty N, et al. Examination of the physical and psychosocial determinants of health behaviour in 4–5-year-old children with congenital cardiac disease. Cardiol Young. 2010; 20:532–537.

16. Linde LM. Psychiatric aspects of congenital heart disease. Psychiatr Clin North Am. 1982; 5:399–406.

17. Reybrouck T, Mertens L, Physical performance and physical activity in grown-up congenital heart disease. Eur J Cardiovasc Prev Rehabil. 2005; 12(5):498-502

18. Thijssen DH, Maiorana AJ, O'Driscoll G, Cable NT, Hopman MT, Green DJ. Impact of inactivity and exercise on the vasculature in humans. Eur J Apply Physiol. 2010; 108:845-875.

For peer review only

For peer review only - http://bmjopen.bmj.com/site/about/guidelines.xhtml

19. Das P, Naylor C, Majeed A. Bringing together physical and mental health within primary care: a new frontier for integrated care. J R Soc Med. 2016; 109:364–6.doi:10.1177/0141076816665270

20. Firth J, Siddiqi N, Koyanagi Ai, Siskind D, Rosenbaum S, Galletly C, et al. The Lancet Psychaitry Comission: A Blueprint for Protecting Physical Health in People with Mental Illness. The Lancet Psychiatry. 2019; 6(8),675-712.

21. National Health Service (NHS). The NHS Long Term Plan. 2019. Available from: https://www.longtermplan.nhs.uk/wp-content/uploads/2019/08/nhs-long-term-plan-version-1.2.pdf

22. National Institute for Health and Care Excellence (NICE). Chronic heart failure in adults: diagnosis and management. NICE guideline 106. 2018. Retrieved from: https://www.nice.org.uk/guidance/ng106

23. British Association for Cardiovascular Prevention and Rehabilitation (BACPR). BACPR Standards and Core Components (3rd Edition). 2017. Retrieved From: http://www.bacpr.com/resources/6A7_BACR_Standards_and_Core_ Components_2017.pdf

24. Akamagwuna, U., Badaly, D. Pediatric Cardiac Rehabilitation: a Review. Curr Phys Med Rehabil Rep. 2019; 7:67–80. https://doi.org/10.1007/s40141-019-00216-9

25. Anderson L, Thompson DR, Oldridge N, Zwisler AD, Rees K, Martin N, et al. Exercise-based cardiac rehabilitation for coronary heart disease. Cochrane Database Syst Rev, 2016; 1:CD001800. doi: 10.1002/14651858.CD001800.pub3

26. Gomes-Neto M, Saquetto MB, Silva CM, Conceicao CS, Carvalho VO. Impact of Exercise Training in Aerobic Capacity and Pulmonary Function in Children and Adolescent After Congenital Heart Disease Surgery: A Systematic Review with Meta-analysis. Pediatr Cardiol. 2016; 37(2):217-24.

27. Duppen N, Takken T, Hopman MTE, Ten Harkel ADJ, Dulfer K, Utens EMWJ, et al. Systematic review of the effects of physical exercise training programmes in children and young adults with congenital heart disease. Int Jnl Cardiology. 2013; 168(3):1779-87.

For peer review only - http://bmjopen.bmj.com/site/about/guidelines.xhtml

28. Tikkanen AU, Oyaga AR, Riano OA, Alvaro EM, Rhodes J. Paediatric cardiac rehabilitation in congenital heart disease: a systematic review. Cardiology in the young. 2012; 22:241-250.

29. Tesson S, Butow PN, Sholler GF, Sharpe L, Kovacs AH, Kasparian NA. Psychological interventions for people affected by childhood-onset heart disease: A systematic review. Health Psychology. 2019; 38(2),151–161. Available from: doi:10.1037/hea0000704

30. Lee S, Lee J, Choi JY. The effect of a resilience improvement program for adolescents with complex congenital heart disease. European Journal of Cardiovascular Nursing, 2017; 16:290–298. Available from: http://dx.doi.org/10.1177/1474515116659836

31. Freedenberg VA, Hinds PS, Friedmann E. Mindfulness-based stress reduction and group support decrease stress in adolescents with cardiac diagnoses: A randomized two-group study. Pediatric Cardiology. 2017; 38:1415–1425. Available from: doi:10.1007/s00246-017-1679-5

32. Capobianco L, Adewusi J, Cooper B, Belcher A, Wells A. Effectiveness of physical and mental health interventions for young people with heart conditions: protocol for a systematic review and meta-analysis. BMJ open. 2023 Feb 1, 13(2):e067342.

33. Wells, A. Metacognitive Therapy for Anxiety and Depression. 2009. New York, NY; London: Guilford Press.

34. Wells A, Reeves D, Capobianco, L. Heal C, Davies L, Heagerty AM, Doherty P, Fisher PL. The effectiveness of psychological interventions for depression and anxiety in cardiac rehabilitation: PATHWAY- a single-blind parallel randomised controlled trial of group metacognitive therapy. Circulation. 2021, 144(1), 23-33. Available from: https://doi.org/10.1161/CIRCULATIONAHA.120.052428

35. Normann N, Morina N. The Efficacy of Metacognitive Therapy: A Systematic Review and Meta-Analysis. Front Psychol. 2018, 14(9),2211. Available from: doi:10.3389/fpsyg.2018.02211

36. Tran D, Maiorana A, Ayer J, Lubans DR, Davis GM, Celermajer DS, d'Udekem Y, Cordina R. Recommendations for exercise in adolescents and adults with congenital heart disease. Progress in cardiovascular diseases. 2020 May 1, 63(3):350-66.

For peer review only

For peer review only - http://bmjopen.bmj.com/site/about/guidelines.xhtml

37. Hirth A, Reybrouck T, Bjarnason-Wehrens B, Lawrenz W, Hoffmann A. Recommendations for participation in competitive and leisure sports in patients with congenital heart disease: a consensus document. Eur J Cardiovasc Prev Rehabil. 2006 Jun; 13(3):293-9. Available from: doi:10.1097/01.hjr.0000220574.22195.d6.

38. Takken T, Giardini A, Reybrouck T, Gewillig M, Hovels-Gurich HH, Longmuir PE, et al. Recommendations for physical activity, recreation sport, and exercise training in paediatric patients with congenital heart disease: a report from the Exercise, Basic & Translational Research Section of the European Association of Cardiovascular Prevention and Rehabilitation, the European Congenital Heart and Lung Exercise Group, and the Association for European Paediatric Cardiology. European Journal of Preventive Cardiology. 2012 Oct, 19(5),1034–1065. Available from: doi:10.1177/1741826711420000.

39. Longmuir PE, Brothers JA, de Ferranti SD, Hayman LL, Van Hare GF, Matherne GP, et al. American Heart Association Atherosclerosis, Hypertension and Obesity in Youth Committee of the Council on Cardiovascular Disease in the Young. Promotion of physical activity for children and adults with congenital heart disease: a scientific statement from the American Heart Association. Circulation. 2013 May 28, 127(21):2147-59. Available from: doi:10.1161/CIR.0b013e318293688f.

40. Wells, A., & Matthews, G. *Attention and emotion: A clinical perspective. 1994; Lawrence Erlbaum Associates, Inc.*

41. Wells, A., & Matthews, G. Modelling cognition in emotional disorder: The S-REF model. Behaviour Research and Therapy,1996. 34(11-12), 881–888. https://doi.org/10.1016/S0005-7967(96)00050-2

42. Wells, A. Breaking the cybernetic code: Understanding and treating the human metacognitive control system to enhance mental health. *Frontiers in Psychology, 2019. 10,* Article 2621. https://doi.org/10.3389/fpsyg.2019.02621

43. McPhillips R, Salmon P, Wells A and Fisher P. Qualitative Analysis of Emotional Distress in Cardiac Patients From the Perspectives of Cognitive Behavioral and Metacognitive Theories: Why Might

For peer review only

For peer review only - http://bmjopen.bmj.com/site/about/guidelines.xhtml

Cognitive Behavioral Therapy Have Limited Benefit, and Might Metacognitive Therapy Be More Effective? Front. Psychol. 2019; 9:2288. doi: 10.3389/fpsyg.2018.02288

44. Schulz K, Altman D, Moher D. CONSORT 2010 statement: updated guidelines for reporting parallel group randomised trials. BMJ. 2010 Mar 23, 340(1):c332. Available from: doi:10.1136/bmj.c332.

45. Eldridge S, Lancaster G, Campbell M, Thabane L, Hopewell S, Coleman C, et al. Defining feasibility and pilot studies in preparation for randomised controlled trials: development of a conceptual framework. PLoS One. 2016; 11(3):e0150205. Available from: doi:10.1371/journal.pone.0150205.

46. Chan A, Tetzlaff J, Altman D, Laupacis A, Gøtzsche P, Krleža-Jerić K, et al. SPIRIT 2013 statement: defining standard protocol items for clinical trials. Ann Int Med. 2013, 158(3):200. Available from: doi:10.7326/0003-4819-158-3-201302050-00583.

47. Chan A, Tetzlaff J, Gotzsche P, Altman D, Mann H, Berlin J, et al. SPIRIT 2013 explanation and elaboration: guidance for protocols of clinical trials. BMJ. 2013 Jan 8, 346(15):e7586. Available from: doi:10.1136/bmj.e7586.

48. Sealed Envelope Ltd. Create a blocked randomisation list [online]. 2022 [cited 1 July 2023]. Available from https://www.sealedenvelope.com/simple-randomiser/v1/lists

49. Goodman R. The Strengths and Difficulties Questionnaire: A Research Note. Journal of Child Psychology and Psychiatry. 1997, 38:581-586. Available from doi:10.1111/j.1469-7610.1997.tb01545.x

50. Muris P, Meesters C, van den Berg F. The Strengths and Difficulties Questionnaire (SDQ). European Child & Adolescent Psychiatry. 2003, 12(1):1-8. Available from: doi:10.1007/s00787-003-0298-2

51. Varni JW, Seid M, Kurtin PS. PedsQL 4.0: Reliability and Validity of the PEdiatric Quality of Life Inventory Version 4.0 Generic Core Scales in Health and Patient Populations. Medical Care. 2001, 39(8):800-812.

For peer review only - http://bmjopen.bmj.com/site/about/guidelines.xhtml

52. Abda A, Bolduc ME, Tsimicalis A, Rennick J, Vatcher D, Brossard-Racine M. Psychosocial outcomes of children and adolescents with severe congenital heart defect: a systematic review and meta-analysis. J Pediatr Psychol. 2019, 44:463-477.

53. den Boer SL, Flipse DH, van der Meulen MH, Backx APCM, du Marchie Sarvaas GJ, Ten Harkel ADJ, et al. Six-Minute Walk Test as a Predictor for Outcome in Children with Dilated Cardiomyopathy and Chronic Stable Heart Failure. Pediatr Cardiol. 2017, 38(3):465-471. Available from: doi:10.1007/s00246-016-1536-y

54. Singh SJ, Morgan MD, Scott S, Walters D, Hardman AE. Development of a shuttle walking test of disability in patients with chronic airways obstruction. Thorax. 1992 Dec, 47(12):1019-24. Available from: doi:10.1136/thx.47.12.1019.

55. Cartwright-Hatton S, Mather A, Illingworth V, Brocki J, Harrington R, Wells A. Development and preliminary validation of the Meta-cognitions Questionnaire—Adolescent Version. Journal of Anxiety Disorders. 2004, 18(3):411-422. Available from: doi:10.1016/S0887-6185(02)00294-3

56. Myers S, Solem S, Wells A. The Metacognitions Questionnaire and Its Derivatives in Children and Adolescents: A Systematic Review of Psychometric Properties. Frontiers in Psychology. 2019 Sep 4, 10,1871. Available from: doi: 10.3389/fpsyg.2019.01871

57. Stevens K. Valuation of the Child Health Utility 9D Index. *PharmacoEconomics.* 2012, 30(8):729-747. Available from: doi:10.2165/11599120-000000000-00000

58. Lindvall K, Vaezghasemi M, Feldman I, Ivarsson A, Stevens K, Petersen S. Feasibility, reliability and validity of the health-related quality of life instrument Child Health Utility 9D (CHU9D) among school-aged children and adolescents in Sweden. Health and Quality of Life Outcomes. 2021, 19(1),193. Available from: doi:10.1186/s12955-021-01830-9

59. Lancaster GA, Dodd S, Williamson PR. Design and analysis of pilot studies: recommendations for good practice. J Eval Clin Pract. 2004, 10(2):307-312. Available from: doi:10.1111/j..2002.384.doc.x

For peer review only

60. Thijssen DH, Maiorana AJ, O'Driscoll G, Cable NT, Hopman MT, Green DJ. Impact of inactivity and exercise on the vasculature in humans. Eur J Apply Physiol. 2010, 108:845-875.

61. Malterud K, Siersma VD, Guassora AD. Sample Size in Qualitative Interview Studies: Guided by Information Power. Qualitative Health Research. 2016, 26(13):1753-1760. Available from: doi:10.1177/1049732315617444

62. Braun, V., & Clarke, V. Successful qualitative research: A practical guide for beginners. 2013. London: Sage.

63. Braun V, Clarke V. Using thematic analysis in psychology. Qual Res Psychol. 2006, 3(2):77–101.

64. Sekhon M, Cartwright M, Francis JJ. Acceptability of healthcare interventions: an overview of reviews and development of a theoretical framework. BMC Health Serv Res. 2017 Jan 26, 17(1),88. Available from: https://doi.org/10.1186/s12913-017-2031-8

65. Khan KA, Petrou S, Rivero-Arias O, Walters SJ, Boyle SE. Mapping EQ-5D utility scores from the PedsQL™ generic core scales. Pharmacoeconomics. 2014, 32(7),693-706.

66. Sweeney R, Chen G, Gold L, Mensah F, Wake M. Mapping PedsQL TM scores onto CHU9D utility scores: estimation, validation and a comparison of alternative instrument versions. Quality of Life Research. 2020, 29(3),639-652.

67. NHS England. New Review of Congenital Heart Services. 2013. Accessed From: https://www.england.nhs.uk/wp-content/uploads/2013/07/180713-item13.pdf

68. Shields GE, Wells A, Doherty P, Heagerty A, Buck D, Davies LM. Cost-effectiveness of cardiac rehabilitation: a systematic review. Heart. 2018, 104,1403–10. Available from: doi:10.1136/heartjnl-2017-312809

69. NHS England. Adult Congenital Heart Disease (ACHD) Specification. 2016. Accessed From: https://www.england.nhs.uk/wp-content/uploads/2018/08/Congenital-heart-disease-standards-and-specifications.pdf

70. Medical Research Council. Mental Health Research Goals 2020-2030. Available from: https://acmedsci.ac.uk/file-download/63608018

For peer review only

For peer review only - http://bmjopen.bmj.com/site/about/guidelines.xhtml

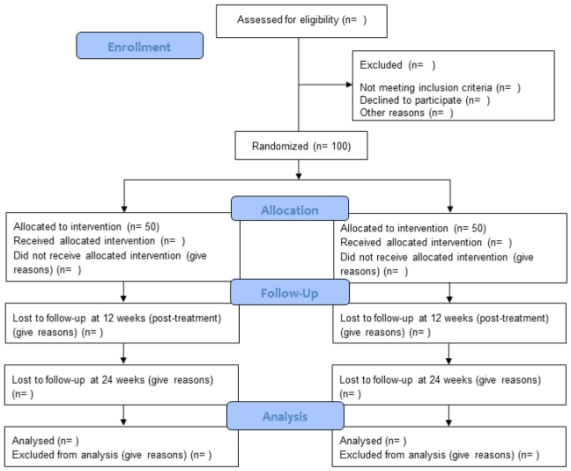

Figure 1. Trial Flow

338x190mm (96 x 96 DPI)

	STUDY PERIOD						
	**Enrolment**	**Baseline**	**Allocation**	**Post-allocation (weeks)**			
**TIMEPOINT**	**Before starting CR**			***1***	***10***	***12***	***24***
**ENROLMENT:**							
**Eligibility screen**	X						
**Informed consent**	X						
**Allocation**			X				
**INTERVENTIONS:**							
**Usual Care**				◆—	—	—◆	
**CR**				◆—	—	—◆	
**ASSESSMENTS:**							
Demographic Information Questionnaire (P)		X				X	X
SDQ (P)		X				X	X
PedsQol (CYP)		X				X	X
6MWT (CYP)		X				X	X
ISWT (CYP)		X				X	X
Physical Activity Monitoring (CYP)		X				X	X
MCQ-A (CYP)		X				X	X
CHU-9D (CYP)		X				X	X
SUQ (P)		X				X	X

For peer review only

Fig. 2 Schedule of enrolment, interventions and assessments. CR, Cardiac Rehabilitation; SDQ, Strength and Difficulties Questionnaire; PedsQol, Paediatric Quality of Life; 6MWT, Six minute walk test; ISWT, Incremental Shuttle Walk Test; MCQ-A, Metacognition Questionnaire-Adolescent; CHU-9D, Child Health Utility-9D; SUQ, Health and Social Care Service-Use Questionnaire; P, parent rated; CYP, child and young person rated

For peer review only - http://bmjopen.bmj.com/site/about/guidelines.xhtml
